# Blinded and unblinded sample size reestimation in crossover trials balanced for period

**DOI:** 10.1002/bimj.201700092

**Published:** 2018-08-03

**Authors:** Michael J. Grayling, Adrian P. Mander, James M. S. Wason

**Affiliations:** ^1^ MRC Biostatistics Unit Cambridge Institute of Public Health Forvie Site, Robinson Way Cambridge UK

**Keywords:** blinded, crossover trial, internal pilot study, sample size reestimation

## Abstract

The determination of the sample size required by a crossover trial typically depends on the specification of one or more variance components. Uncertainty about the value of these parameters at the design stage means that there is often a risk a trial may be under‐ or overpowered. For many study designs, this problem has been addressed by considering adaptive design methodology that allows for the re‐estimation of the required sample size during a trial. Here, we propose and compare several approaches for this in multitreatment crossover trials. Specifically, regulators favor reestimation procedures to maintain the blinding of the treatment allocations. We therefore develop blinded estimators for the within and between person variances, following simple or block randomization. We demonstrate that, provided an equal number of patients are allocated to sequences that are balanced for period, the proposed estimators following block randomization are unbiased. We further provide a formula for the bias of the estimators following simple randomization. The performance of these procedures, along with that of an unblinded approach, is then examined utilizing three motivating examples, including one based on a recently completed four‐treatment four‐period crossover trial. Simulation results show that the performance of the proposed blinded procedures is in many cases similar to that of the unblinded approach, and thus they are an attractive alternative.

## 1 INTRODUCTION

Crossover trials, in which participants are randomly allocated to receive a sequence of treatments across a series of time periods, are an extremely useful tool in clinical research. Their nature permits each patient to act as their own control, exploiting the fact that in most instances the variability of measurements on different subjects in a study will be far greater than that on the same subject. In this way, crossover trials are often more efficient than parallel group trials. Like most experimental designs, the determination of the sample size required by a crossover trial, to achieve a certain power for a particular treatment effect, depends on the significance level, and at least one factor that accounts for the participant's variance in response to treatment. While the former are designated quantities, the variance factors will usually be subject to substantial uncertainty at the design stage. Their value will often be greatly affected by components of the current trial, such as inclusion/exclusion criteria for example, that renders estimates obtained from previous trials biased. This is troubling since sample size calculation is of paramount importance in study design. Planning a trial that is too large results in an unnecessary number of patients being made susceptible to interventions that may be harmful. It also needlessly wastes valuable resources in terms of time, money, and available trial participants. In contrast, too small a sample size confers little chance of success for a trial. The consequences of this could be far reaching: a wrong decision may lead to the halting of the development of a therapy, which could deprive future patients of a valuable treatment option.

To address this problem in a parallel group setting with normally distributed outcome variables, Wittes and Brittain (1990), building upon previous work by Stein (1945), proposed the internal pilot study design. In their approach, at an interim time period the accrued data is unblinded, the within‐group variance computed, and the trial's required sample size adjusted if necessary. However, unblinding an ongoing trial can reduce its integrity and introduce bias (ICH, 1998). Consequently, Gould and Shih (1992) explored several approaches for reestimating the required sample size in a blinded manner. Since then, a number of papers have advocated for reestimation in a parallel group setting to be based upon a crude one‐sample estimate of the variance, and methodology has also been proposed that allows the type‐I error‐rate to be more accurately controlled (Kieser & Friede, 2003). More recently, much work has been conducted on similar methods for an array of possible trial designs and types of outcome variable (see, e.g. Jensen & Kieser, 2010; and Togo & Iwasaki, 2011), with these methods also gaining regulatory acceptance (CHMP, 2007; FDA, 2010).

Thus, today, sample size reestimation procedures have established themselves for parallel group trials as an advantageous method to employ when there is pre‐trial uncertainty over the appropriate sample size. In contrast, there has been little exploration of such methodology within the context of multitreatment crossover trials. Golkowski, Friede, and Kieser (2014) recently explored a blinded sample size reestimation procedure for establishing bioequivalence in a trial utilizing an AB/BA crossover design. Jones and Kenward (2014) discussed how the results of Kieser and Friede (2003) could be rephrased for an AB/BA crossover trial testing for superiority. In addition, several unblinded reestimation procedures for AB/BA bioequivalence trials have been proposed (Potvin et al., 2007; Montague et al., 2012; Xu et al., 2016), the performance of which has recently been extensively compared (Kieser & Rauch, 2015). The work of Lake, Kammann, Klar, and Betensky (2002) and van Schie and Moerbeek (2014) on sample size reestimation in cluster randomized trials has some parallels with the methodology required for crossover trials, because of the necessitated mixed model for data analysis. Likewise, this is true of the methodology presented by Zucker and Denne (2002) on reestimation procedures for longitudinal trials. However, we are unaware of any article that explicitly discusses reestimation in crossover trials with more than two‐treatments. There are many examples of such trials in the literature, while they also remain the focus of much research (see, e.g. Bailey & Druilhet, 2014; and Lui & Chang, 2016).

In this article, we consider several possible approaches to the interim reassessment of the sample size required by a multitreatment crossover trial. We assume a normally distributed outcome variable, and that a commonly utilized linear‐mixed model will be employed for data analysis. We focus primarily on a setting in which the final analysis is based on many‐to‐one comparisons for one‐sided null hypotheses, but provide additional guidance for other possibilities in the Supplementary Material. Blinded procedures for estimating the between and within person variance in response are proposed, following either simple or block randomisation to sequences that are balanced for period. The performance of these estimators is contrasted to that of an unblinded procedure via a simulation study motivated by a real four‐treatment four‐period crossover trial. Additionally, in the Supplementary Material we provide results for two additional examples. We now proceed by specifying the notation used in the re‐estimation procedures. Our findings are then summarized in Section 3, before we conclude in Section 4 with a discussion.

## 2 METHODS

### 2.1 Hypotheses, notation, and analysis

We consider a crossover trial with *D* treatments, indexed d=0,⋯,D−1. Treatments d=1,…,D−1 are considered experimental, and are to be compared to the common control d=0. We suppose that *K* sequences, indexed k=1,⋯,K, are utilised for treatment allocation, and denote by $n_{k}$ the number of patients allocated to sequence *k*. The number of periods in the trial, which is equal to the length of each of the sequences, is denoted by *P*.

We restrict our focus to trials with normally distributed outcome data, to be analysed using the following linear‐mixed model

(1) $$y_{ijk}=\mu_{0}+\pi_{j}+\tau_{\text{d}(j,k)}+s_{ik}+\varepsilon_{ijk},\;i=1,\cdots ,n_{k},\;j=1,\cdots ,P,\;k=1,\cdots ,K.$$

Here

(i) $y_{ijk}$ is the response for individual *i*, in period *j*, on sequence *k*;
(ii) μ_0_ is an intercept term; the mean response on treatment 0 in period 1;
(iii) $\pi_{j}$ is a fixed effect for period *j*, with the identifiability constraint $\pi_{1}=0$;
(iv) $\tau_{\text{d}(j,k)}$ is a fixed direct treatment effect for the treatment administered to an individual in period *j*, on sequence *k*, with the identifiability constraint $\tau_{0}=0$. Thus d(j,k)=0,⋯,D−1;
(v) $s_{ik}∼N\left(0,\sigma_{b}^{2}\right)$ is a random effect for individual *i* on sequence *k*;
(vi) $\varepsilon_{ijk}∼N\left(0,\sigma_{e}^{2}\right)$ is the residual for the response from individual *i*, in period *j*, on sequence *k*.

This model, and its implied covariance structure, is the standard for a crossover trial that ignores the possible effects of carryover. Thus we are implicitly heeding the advice of Senn (1992), and others, that a crossover trial should not be conducted when carryover is likely to be an issue. Furthermore, note that by the above, two observations $y_{i_{1}j_{1}k_{1}}$ and $y_{i_{2}j_{2}k_{2}}$ are independent unless $i_{1}=i_{2}$ and $k_{1}=k_{2}$.

We assume that the following hypotheses are to be tested, to attempt to establish the superiority of each experimental intervention versus the control

$$H_{0d}:\tau_{d}\leq 0,\;H_{1d}:\tau_{d}>0,\;d=1,\cdots ,D-1.$$

Note though that for Examples 1 and 3, slightly different hypotheses are assessed, as negative effects imply efficacy. Additionally, in the Supplementary Material we detail how one can handle alternate hypotheses of interest.

We suppose that it is desired to strongly control the FWER, the maximal probability of one or more incorrect rejections among the family of null hypotheses for all possible treatment effects, to some specified level α∈(0,1). There are several possible ways to define power in a multitreatment setting. Throughout, we assume that pairwise power of at least 1−β∈(0,1) to reject, without loss of generality, *H*_01_ is required when $\tau_{1}=\delta >0$ for designated type‐II error‐rate β and clinically relevant difference δ. Thus, from here, when referring to power we mean the probability that *H*_01_ is rejected. However, in the Supplementary Material we describe how a desired familywise power could be achieved.

To test the hypotheses, we assume that *N* patients in total will be recruited to the trial, with each randomized to one of the *K* sequences, and that the the linear‐mixed model (1) will be fitted to the accumulated data. Note that in fitting this model, a choice must be made over whether to utilize maximum likelihood, or restricted error maximum likelihood (REML), estimation. Given the bias of the maximum likelihood estimator of the variance components of a linear‐mixed model in finite samples, and that crossover trials are often conducted with relatively small sample sizes, here we always take the latter approach. Note though that this would have little effect for larger sample sizes. For further details on these considerations, we refer the reader to, for example, Fitzmaurice, Laird, & Ware (2011). In brief, the REML estimation procedure, for a linear‐mixed model of the form y=Xβ+Zb+ε with b∼N(0,G) and ε∼N(0,R), iteratively optimizes the parameter estimates for the effects in the model. The following modified log‐likelihood is maximized to provide an estimate, $\hat{\Sigma }$, for $\Sigma =ZGZ^{⊤}+R$, using an estimate, $\hat{\beta }$, for ***β***

$$-\frac{1}{2}\log\left|\Sigma \right|-\frac{1}{2}{\left(y-X\hat{\beta }\right)}^{⊤}\Sigma^{-1}\left(y-X\hat{\beta }\right)-\frac{1}{2}\log|X^{⊤}\Sigma^{-1}X|.$$

Then, $\hat{\beta }$ is updated to

$$\hat{\beta }={\left(X^{⊤}{\hat{\Sigma }}^{-1}X\right)}^{-1}X^{⊤}{\hat{\Sigma }}^{-1}y,$$

and the process repeated. Given the final solutions $\hat{\beta }$ and $\hat{\Sigma }$, we take $\text{Var}\left(\hat{\beta }\right)={\left(X^{⊤}{\hat{\Sigma }}^{-1}X\right)}^{-1}$.

In our case, $\beta ={\left(\mu_{0},\pi_{2},\cdots ,\pi_{P},\tau_{1},\cdots ,\tau_{D-1}\right)}^{⊤}$, and the following D−1 Wald test statistics are formed

$$T_{d}=\frac{{\hat{\tau }}_{d}}{\sqrt{\text{Var}({\hat{\tau }}_{d})}},\;d=1,\cdots ,D-1,$$

where ${\hat{\tau }}_{d}$ and $\text{Var}({\hat{\tau }}_{d})$ are extracted from $\hat{\beta }$ and $\text{Var}(\hat{\beta })$, respectively.

Next, we reject $H_{0d}$ if $T_{d}>e$, with *e* chosen to control the FWER. Explicitly, using a Dunnett test (Dunnett, 1955), we take *e* as the solution to

(2) $$1-\alpha =\Psi_{D-1}\left\{{\left(e,\cdots ,e\right)}^{T},\text{Var}\left(T\right),\nu_{N}\right\},$$

where $\Psi_{M}\left\{x,\Lambda ,\nu \right\}$ is the *M*‐dimensional cumulative distribution function of a central multivariate *t*‐distribution with covariance matrix Λ and ν degrees of freedom. We take the degrees of freedom here, for sample size *N*, to be $\nu_{N}=\left(N-1\right)\left(P-1\right)\;-\;\left(D-1\right)$, which arises from that associated with an analogous multilevel ANOVA design. Moreover, Var(T) is the covariance matrix of $T={\left(T_{1},\cdots ,T_{D-1}\right)}^{⊤}$, which can be calculated using $\text{Var}(\hat{\beta })$.

Now, in this case, if $\sigma_{e}^{2}$ and $\sigma_{b}^{2}$ were known, and we assumed that $n_{1}=\cdots =n_{K}$, we could derive a simple formula for the total number of patients, *N*, required to achieve the desired power for the trial. Here, we denote this formula using the function $\text{N}(\sigma_{e}^{2},\sigma_{b}^{2})$, explicitly stating its dependence upon the within and between person variances. In the Supplementary Material, we elaborate on how this formula can be derived.

Our problem, as discussed, is that in practice $\sigma_{e}^{2}$ and $\sigma_{b}^{2}$ are rarely known accurately at the design stage. Therefore, we propose to reestimate the required sample size at an interim analysis timed after $n_{\text{int}}\in N$ patients. That is, we consider several methods to construct estimates, ${\hat{\sigma }}_{e}^{2}$ and ${\hat{\sigma }}_{b}^{2}$, for $\sigma_{e}^{2}$ and $\sigma_{b}^{2}$ , respectively, based on the data accrued up to the interim analysis. Then, the final sample size for the trial is taken as

$$\hat{N}=\left\{\begin{array}{cc} n_{\text{int}} & \text{if}\;\text{N}\left({\hat{\sigma }}_{e}^{2},{\hat{\sigma }}_{b}^{2}\right)\leq n_{\text{int}},\\ \left\lceil \text{N}\left({\hat{\sigma }}_{e}^{2},{\hat{\sigma }}_{b}^{2}\right)\right\rceil & \text{if}\;n_{\text{int}}<\text{N}\left({\hat{\sigma }}_{e}^{2},{\hat{\sigma }}_{b}^{2}\right)<n_{\text{max}},\\ n_{\text{max}} & \text{if}\;\text{N}\left({\hat{\sigma }}_{e}^{2},{\hat{\sigma }}_{b}^{2}\right)\geq n_{\text{max}}, \end{array}\right.$$

where ⌈x⌉ denotes the nearest integer greater than or equal to *x* and $n_{\text{max}}\in N$ is a specified maximal allowed sample size. It could be based, for example, on the cost restrictions or feasible recruitment rate of a trial. Of course, if $\hat{N}=n_{\text{max}}$ then the trial will be expected to be underpowered. Thus, if necessary, additional patients are recruited and a final analysis conducted as above based on the calculated values of the test statistics $T_{d}$, and the critical value *e* as defined in Equation (2).

Throughout, to give our function N(·) a simple form, we consider values of $n_{\text{int}}$ that imply an equal number of patients could be allocated to each of the *K* sequences, and assume randomisation schemes that ensure this is the case. Moreover, for reasons to be elucidated shortly, we consider from here only settings where the *K* sequences are balanced for period. That is, across the chosen sequences, each treatment appears an equal number of times in each period. We now proceed by detailing each of our explored methods for estimating $\sigma_{e}^{2}$ and $\sigma_{b}^{2}$ based on the internal pilot data.

### 2.2 Unblinded estimator

The first of the methods we consider is an unblinded procedure. As noted, such an approach is typically less well favored by regulatory agencies. However, though this may not always actually prove to be the case (see, e.g. Friede & Kieser, 2013), one may anticipate its performance in terms of estimating the key variance components and provided desired operating characteristics to be preferable to that of the blinded procedures. This method therefore serves as a standard against which to assess the blinded estimators. Explicitly, this approach breaks the randomization code and fits the linear‐mixed model (1) to the accrued data using REML estimation. With the REML estimates of $\sigma_{e}^{2}$ and $\sigma_{b}^{2}$ obtained, they are utilized in the reestimation procedure as described above.

### 2.3 Adjusted blinded estimator

Zucker, Wittes, Schabenberger, and Brittan (1999) considered a blinded estimator for two‐arm parallel trial designs based on an adjustment to the one‐sample variance. Golkowski et al. (2014) considered a similar unadjusted procedure for two‐arm bioequivalence trials. Here, we consider a similar approach for multi‐treatment crossover trials. Specifically, the following blinded estimators of the within and between person variances are used

$$\begin{array}{cc} {\hat{\sigma }}_{e}^{2} & =\frac{1}{2\left(P-1\right)\left(n_{\text{int}}-1\right)}\sum_{j=2}^{P}\sum_{k=1}^{K}\sum_{i=1}^{n_{\text{int}}/K}{\left(p_{ijk}-{\bar{p}}_{j}\right)}^{2}+\\ & \;-\frac{n_{\text{int}}}{2K\left(P-1\right)\left(n_{\text{int}}-1\right)}\sum_{j=2}^{P}\sum_{k=1}^{K}{\left(\tau_{\text{d}(j,k)}^{*}-\tau_{\text{d}(j-1,k)}^{*}\right)}^{2},\\ {\hat{\sigma }}_{b}^{2} & =\frac{1}{2}\left\{\frac{1}{2\left(P-1\right)\left(n_{\text{int}}-1\right)}\sum_{j=2}^{P}\sum_{k=1}^{K}\sum_{i=1}^{n_{\text{int}}/K}{\left(q_{ijk}-{\bar{q}}_{j}\right)}^{2}-{\hat{\sigma }}_{e}^{2}+\right.\\ & \;\;\left.-\frac{n_{\text{int}}}{2K\left(P-1\right)\left(n_{\text{int}}-1\right)}\sum_{j=2}^{P}\sum_{k=1}^{K}{\left(\tau_{\text{d}(j,k)}^{*}+\tau_{\text{d}(j-1,k)}^{*}\right)}^{2}+\right.\\ & \;\;\;\left.+\frac{2n_{\text{int}}}{D^{2}\left(n_{\text{int}}-1\right)}{\left(\sum_{k=1}^{K}\tau_{\text{d}(1,k)}\right)}^{2}\right\}, \end{array}$$

for specified $\tau_{d}^{*}$, d=0,⋯,D−1, with $\tau_{0}^{*}=0$, where

$$\begin{array}{cc} p_{ijk} & =y_{ijk}-y_{ij-1k},\\ q_{ijk} & =y_{ijk}+y_{ij-1k},\\ {\bar{p}}_{j} & =\frac{1}{n_{\text{int}}}\sum_{k=1}^{K}\sum_{i=1}^{n_{\text{int}}/K}p_{ijk},\\ {\bar{q}}_{j} & =\frac{1}{n_{\text{int}}}\sum_{k=1}^{K}\sum_{i=1}^{n_{\text{int}}/K}q_{ijk}. \end{array}$$

In the Supplementary Material, we show that if $\tau_{d}^{*}=\tau_{d}$ for d=1,⋯,D−1 then $\text{E}\left({\hat{\sigma }}_{e}^{2}\right)=\sigma_{e}^{2}$ and $\text{E}\left({\hat{\sigma }}_{b}^{2}\right)=\sigma_{b}^{2}$, and thus ${\hat{\sigma }}_{e}^{2}$ and ${\hat{\sigma }}_{b}^{2}$ are unbiased estimators for $\sigma_{e}^{2}$ and $\sigma_{b}^{2}$ , respectively. This is the reason for our restrictions on the employed randomization scheme (which assumes $n_{1}=\cdots =n_{K}=n_{\text{int}}/K$ at the interim reassessment), and the employed sequences (which are assumed to be balanced for period). The above estimator could be used when there is imbalance in the number of patients allocated to each sequence, or without making this restriction on the sequences, but results on the expected values of the variance components would have a more complex form. It is therefore advantageous to ensure an equal number of patients are allocated to each sequence, and also logical to utilize period‐balanced sequences. We also view it as sensible therefore to explore the performance of the estimators in this case.

It is also important to assess the sensitivity of the performance of these estimators to the choice of the $\tau_{d}^{*}$, hoping for it to have negligible impact as in analogous procedures for other trial settings (Kieser & Friede, 2002). Adapting previous works (see, e.g. Kieser & Friede, 2003; Zucker et al., 1999; Gould & Shih, 1992), we assess this procedure for $\tau_{d}^{*}=0$, and $\tau_{d}^{*}=\delta$, d=1,⋯,D−1, and refer to these henceforth as the null adjusted and alternative adjusted reestimation procedures, respectively.

Note that one limitation of this approach in practice is that there is no guarantee that the above value for ${\hat{\sigma }}_{b}^{2}$ will be positive. Therefore, we actually reevaluate the required sample size as $\text{N}\{{\hat{\sigma }}_{e}^{2},\max\left(0,{\hat{\sigma }}_{b}^{2}\right)\}$. For the examples provided in the Supplementary Material, we demonstrate that the above procedure still performs well despite this inconvenience. Moreover, in certain routinely faced scenarios, as will be discussed shortly, the value of $\sigma_{b}^{2}$ is inconsequential and this issue therefore no longer exists. However, in general this must be kept in mind when considering using this procedure for sample size reestimation.

### 2.4 Blinded estimator following block randomization

The above reestimation procedures are explored within the context of a simple randomisation scheme that only ensures an equal number of patients are allocated to each sequence prior to the interim reassessment. In contrast, the final blinded estimator we consider exploits the advantages block randomization can bring, extending the methodology presented in Xing and Ganju (2005) for parallel arm trials to crossover studies.

We suppose that patients are allocated to sequences in *B* blocks, each of length $n_{B}$ (with these values chosen such that $Bn_{B}=n_{\text{int}}$). We recategorize our data as $y_{ijb}$, the response from patient $i=1,\cdots ,n_{B}$, in period *j*, in block *b*. Then, the following blinded estimators are used to recalculate the required sample size

$$\begin{array}{cc} {\hat{\sigma }}_{e}^{2} & =\frac{1}{2\left(P-1\right)\left(n_{\text{int}}-B\right)}\sum_{j=2}^{P}\sum_{b=1}^{B}\sum_{i=1}^{n_{B}}{\left(p_{ijb}-{\bar{p}}_{jb}\right)}^{2},\\ {\hat{\sigma }}_{b}^{2} & =\frac{1}{2}\left\{\frac{1}{2\left(P-1\right)\left(n_{\text{int}}-B\right)}\sum_{j=2}^{P}\sum_{b=1}^{B}\sum_{i=1}^{n_{B}}{\left(q_{ijb}-{\bar{q}}_{jb}\right)}^{2}-{\hat{\sigma }}_{e}^{2}\right\}, \end{array}$$

where

$$\begin{array}{cc} p_{ijb} & =y_{ijb}-y_{ij-1b},\\ q_{ijb} & =y_{ijb}+y_{ij-1b},\\ {\bar{p}}_{jb} & =\frac{1}{n_{B}}\sum_{i=1}^{n_{B}}p_{ijb},\\ {\bar{q}}_{jb} & =\sum_{i=1}^{n_{B}}\sum_{i=1}^{n_{B}}q_{ijb}. \end{array}$$

In the Supplementary Material, provided that an equal number of patients are allocated to each of a set of period balanced sequences, these are also shown to be unbiased estimators for $\sigma_{e}^{2}$ and $\sigma_{b}^{2}$. Note though that as above, we must actually reestimate *N* using $\text{N}\{{\hat{\sigma }}_{e}^{2},\max\left(0,{\hat{\sigma }}_{b}^{2}\right)\}$. Additionally, when using block randomization, the actual sample size used by a trial may differ from $\hat{N}$, if it is not divisible by the block length $n_{B}$.

## 3 SIMULATION STUDY

### 3.1 Motivating examples

We present results for three motivating examples based on real crossover trials. Example 1 is described in Section 3.2, with Examples 2 and 3 discussed in the Supplementary Material, where their associated results are also presented. Among the three examples we consider settings with a range of required sample sizes, utilising complete block, incomplete block, and extra‐period designs. This allows us to provide a thorough depiction of the performance of the various estimators in a wide range of realistic trial design settings.

R (R Core Team, 2016) source code to reproduce our results is available as Supporting Information on the journal's web page (http://onlinelibrary.wiley.com/doi/10.1002/bimj.201700092/suppinfo).

### 3.2 Example 1: TOMADO

First, we assess the performance of the various reestimation procedures using the TOMADO trial as motivation. TOMADO compared the clinical effectiveness of a range of mandibular devices for the treatment of obstructive sleep‐apnea hypopnea. Precise details can be found in Quinnell et al. (2014). Briefly, TOMADO was a four‐treatment four‐period crossover trial, with patients allocated treatment sequences using two Williams squares. The data for the outcome Epworth Sleepiness Scale was to be analyzed using linear‐mixed model (1), with the following hypotheses tested

$$H_{0d}:\tau_{d}\geq 0,\;H_{1d}:\tau_{d}<0,\;d=1,\cdots ,D-1,$$

since a reduction in the Epworth Sleepiness Scale score is indicative of an efficacious treatment. Consequently, the null hypotheses were to be rejected if $T_{d}<-e$, using the value of *e* determined as above.

Following the methodology described in the Supplementary Material, we can demonstrate that when complete‐block period‐balanced sequences are used for treatment allocation, that the required sample size has no dependence upon the between person variance $\sigma_{b}^{2}$. Explicitly, we have

$$\text{N}\left(\sigma_{e}^{2},\sigma_{b}^{2}\right)\equiv \text{N}\left(\sigma_{e}^{2}\right)=\frac{2\sigma_{e}^{2}{\left(z_{1-\alpha_{*}}+z_{1-\beta }\right)}^{2}}{\delta^{2}},$$

where $\alpha_{*}$ is defined in the Supplementary Material. See Jones and Kenward (2014), for an alternative derivation of this formula. This substantially simplifies the reestimation procedure, as we only need to provide a value for $\sigma_{e}^{2}$, and do not require use of the estimators for $\sigma_{b}^{2}$.

TOMADOs complete case analysis estimated the following values for the various components of the linear‐mixed model (1)

$${\hat{\mu }}_{0}=10.65,\;{\hat{\pi }}_{2}=-0.77,\;{\hat{\pi }}_{3}=-0.96,\;{\hat{\pi }}_{4}=-0.55,$$

$${\hat{\tau }}_{1}=-1.51,\;{\hat{\tau }}_{2}=-2.15,\;{\hat{\tau }}_{3}=-2.37,\;{\hat{\sigma }}_{e}^{2}=6.51,\;{\hat{\sigma }}_{b}^{2}=10.12.$$

Therefore, for $\sigma_{e}^{2}={\hat{\sigma }}_{e}^{2}$, the trials planned recruitment of 72 patients would have conferred power of 0.8 at a significance level of 0.05 for δ=−1.24. Consequently, we set β=0.2 and α=0.05 throughout. In the main manuscript, we additionally take δ=−1.24 and $\sigma_{b}^{2}=10.12$ always. The effect of other underlying values for δ and $\sigma_{b}^{2}$ is considered in the Supplementary Material. In contrast, whilst we focus here on the case with $\sigma_{e}^{2}=6.51$, we also consider the influence of alternative values for this parameter. When simulating data we take $\mu_{0}=10.65$, $\pi_{2}=-0.77$, $\pi_{3}=-0.96$, and $\pi_{4}=-0.55$. However, the effect of other period effects is discussed in Section 4 and in the Supplementary Material.

We explore the performance of the procedures under the global null hypothesis ($\tau_{1}=\tau_{2}=\tau_{3}=0$), when only treatment one is effective ($\tau_{1}=\delta ,\tau_{2}=\tau_{3}=0$), when treatments one and two are effective ($\tau_{1}=\tau_{2}=\delta ,\tau_{3}=0$), under the global alternative hypothesis ($\tau_{1}=\tau_{2}=\tau_{3}=\delta$), and under what we refer to henceforth as the observed treatment effects ($\tau_{1}=-1.51,\tau_{2}=-2.15,\tau_{3}=-2.37$). For simplicity, we assume a single Latin square was used for treatment allocation, and set $n_{\text{max}}=$ 1,000 so that there is no practical upper limit on the allowed sample size. In all cases, the average result for a particular design and analysis scenario was determined using 100,000 trial simulations.

### 3.3 Distributions of ${\hat{\sigma }}_{e}^{2}$ and $\hat{N}$

**Figure 1:**
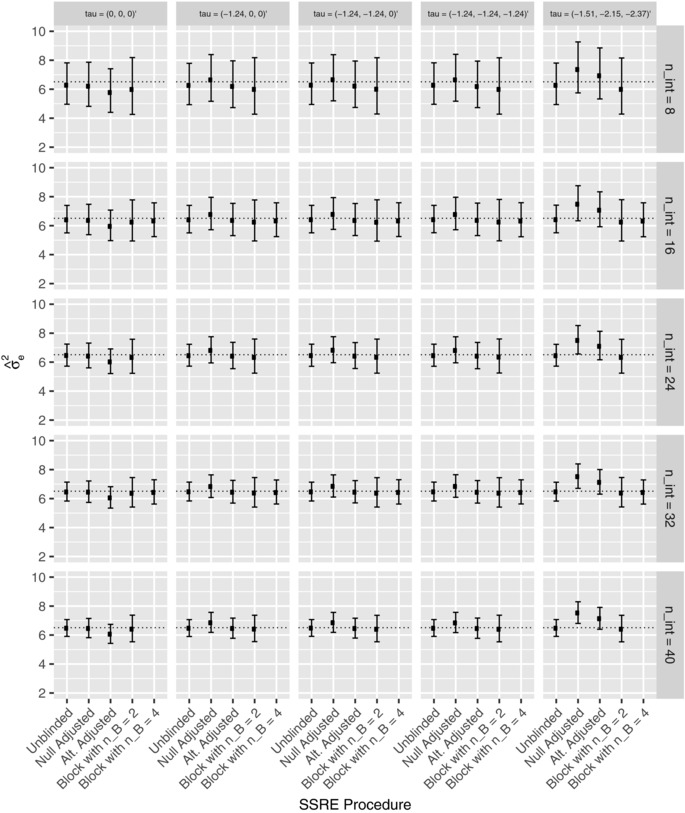
The distribution of ${\hat{\sigma }}_{e}^{2}$ is shown for each of the reestimation procedures for several values of ***τ***, and several values of $n_{\text{int}}$, for Example 1. Precisely, for each scenario, the median, lower, and upper quartile values of ${\hat{\sigma }}_{e}^{2}$ across the simulations are given. The dashed line indicates the true value of $\sigma_{e}^{2}$

**Figure 2:**
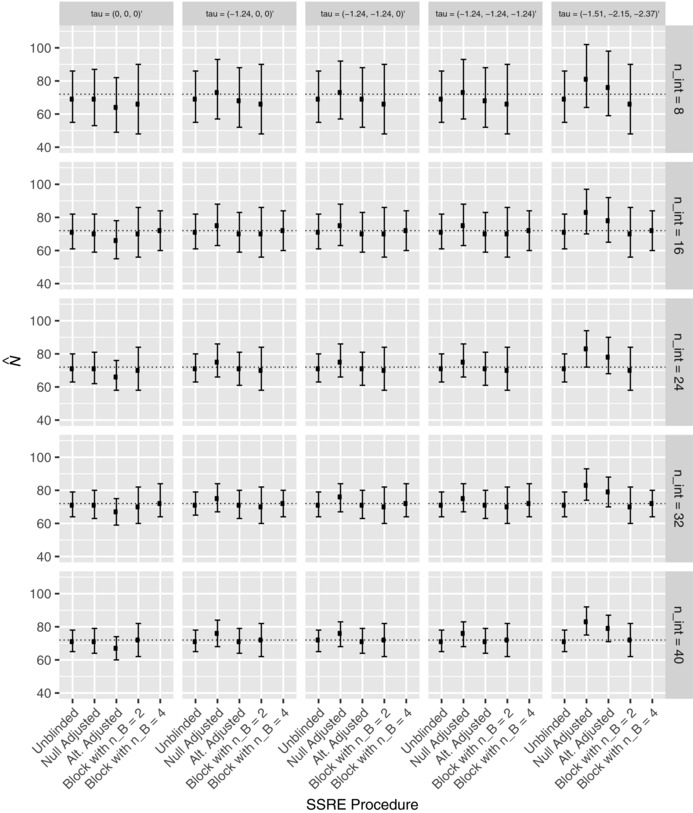
The distribution of $\hat{N}$ is shown for each of the reestimation procedures for several values of ***τ***, and several values of $n_{\text{int}}$, for Example 1. Precisely, for each scenario, the median, lower, and upper quartile values of $\hat{N}$ across the simulations are given. The dashed line indicates the true required value of *N*

First, the performance of the reestimation procedures was explored for the parameters listed in Section 3.2, with $\sigma_{e}^{2}=6.51$, and $n_{\text{int}}\in \left\{8,16,24,32,40\right\}$. The resulting distributions of ${\hat{\sigma }}_{e}^{2}$, the interim estimate of $\sigma_{e}^{2}$, are shown in Figure 1 via the median, lower and upper quartiles in each instance. Additionally, Figure 2 depicts the equivalent results for the distribution of $\hat{N}$, the interim reestimated value for *N*. The results are grouped according to the timing of the reestimation and by the true value of the treatment effects. Note that $n_{B}=4$ is only considered for values of $n_{\text{int}}$ that allows an equal number of patients to be allocated to each sequence by the interim analysis.

The median value of ${\hat{\sigma }}_{e}^{2}$ for the unblinded procedure is always close to, but typically slightly less than, the true value $\sigma_{e}^{2}$. The same statement holds for the block randomization procedure with $n_{B}=2$ or 4. However, while this is true for the adjusted procedures under the global null hypothesis, it is not otherwise always the case. In particular, both perform poorly for the observed treatment effects.

As would be anticipated, the alternative adjusted procedure has lower median values for ${\hat{\sigma }}_{e}^{2}$ than the null adjusted procedure. Moreover, using the block randomised reestimation procedure with $n_{B}=4$ seems to improve performance over $n_{B}=2$, both in terms of the median value of ${\hat{\sigma }}_{e}^{2}$, and by imparting a smaller interquartile range for ${\hat{\sigma }}_{e}^{2}$.

The results for $\hat{N}$ mirror those for ${\hat{\sigma }}_{e}^{2}$. Thus $\hat{N}$ is larger for the adjusted estimators under the observed treatment effects, but otherwise the distributions are comparable.

Increasing the value of $n_{\text{int}}$ reduces the interquartile range for ${\hat{\sigma }}_{e}^{2}$ and $\hat{N}$ for each procedure, and results in median values closer to the truth, as would be expected. Finally, we observe that the interquartile range for the unblinded procedure is often smaller than that of its adjusted or block randomisation counterparts.

### 3.4 Familywise error‐rate and power

For the scenarios from Section 3.3 that were not conducted under the observed treatment effects, the estimated FWER and power were also recorded. The results are displayed in Table 1.

**Table 1:** The estimated familywise error‐rate (FWER) is shown for each of the considered reestimation procedures and several values of $n_{\text{int}}$ under the global null hypothesis, for Example 1

			Power		
Reestimation procedure	$n_{\text{int}}$	FWER	τ=(δ,0,0)	τ=(δ,δ,0)	τ=(δ,δ,δ)
Unblinded	8	0.0513	0.7704	0.7694	0.7687
Null Adjusted	8	0.0496	0.7743	0.7809	0.7753
Alt. Adjusted	8	0.0500	0.7440	0.7512	0.7432
Block rand. with $n_{B}=2$	8	0.0509	0.7443	0.7455	0.7428
Unblinded	16	0.0506	0.7906	0.7893	0.7867
Null Adjusted	16	0.0512	0.7956	0.8010	0.7942
Alt. Adjusted	16	0.0495	0.7702	0.7731	0.7691
Block rand. with $n_{B}=2$	16	0.0512	0.7720	0.7723	0.7747
Block rand. with $n_{B}=4$	16	0.0525	0.7858	0.7887	0.7868
Unblinded	24	0.0509	0.7963	0.7934	0.7950
Null Adjusted	24	0.0496	0.8019	0.8071	0.7990
Alt. Adjusted	24	0.0508	0.7776	0.7793	0.7770
Block rand. with $n_{B}=2$	24	0.0504	0.7821	0.7838	0.7835
Unblinded	32	0.0520	0.7977	0.7962	0.7988
Null Adjusted	32	0.0509	0.8055	0.8109	0.8072
Alt. Adjusted	32	0.0498	0.7772	0.7857	0.7812
Block rand. with $n_{B}=2$	32	0.0514	0.7907	0.7879	0.7887
Block rand. with $n_{B}=4$	32	0.0511	0.8014	0.8002	0.8035
Unblinded	40	0.0516	0.7967	0.8010	0.8000
Null Adjusted	40	0.0504	0.8081	0.8115	0.8062
Alt. Adjusted	40	0.0498	0.7828	0.7858	0.7842
Block rand. with $n_{B}=2$	40	0.0518	0.7914	0.7926	0.7942

Corresponding values of the power when only treatment one is effective, treatments one and two are effective, or under the global alternative hypothesis when all three experimental treatments are effective, are also shown. The Monte Carlo error of the FWER and power values is approximately 0.0007 and 0.0013, respectively in each instance. All figures are given to four decimal places

The FWER for each of the procedures is usually close to the nominal level, with a maximal value of 0.052 for the unblinded procedure with $n_{\text{int}}=32$. The adjusted procedures arguably have the smallest inflation across the considered values of $n_{\text{int}}$.

In most cases the reestimation procedures attain a power close to the desired level. Of the adjusted procedures, the null adjusted has a larger power, as would be anticipated given our observations on ${\hat{\sigma }}_{e}^{2}$ and $\hat{N}$ above. In fact, the null adjusted method conveys the highest power for each value of $n_{\text{int}}$. The power of the block randomized procedures is typically similar to that of the alternative adjusted method. In addition, whether only treatment one, treatments one and two, or all three treatments are effective has little effect on the power.

There is no clear to trend as to the effect of increasing $n_{\text{int}}$ on the FWER, however it leads in almost all instances to an improvement in power. Finally, increasing the value for $n_{B}$ in the block randomization procedure increases power as would be predicted.

### 3.5 Influence of $\sigma_{e}^{2}$

In this section, we consider the influence of the value of $\sigma_{e}^{2}$ on the performance of our reestimation procedures. Specifically, while we know that increasing $\sigma_{e}^{2}$ will increase the required sample size, we would like to assess the effect this has upon the ability of the methods to control the FWER and attain the desired power.

**Figure 3:**
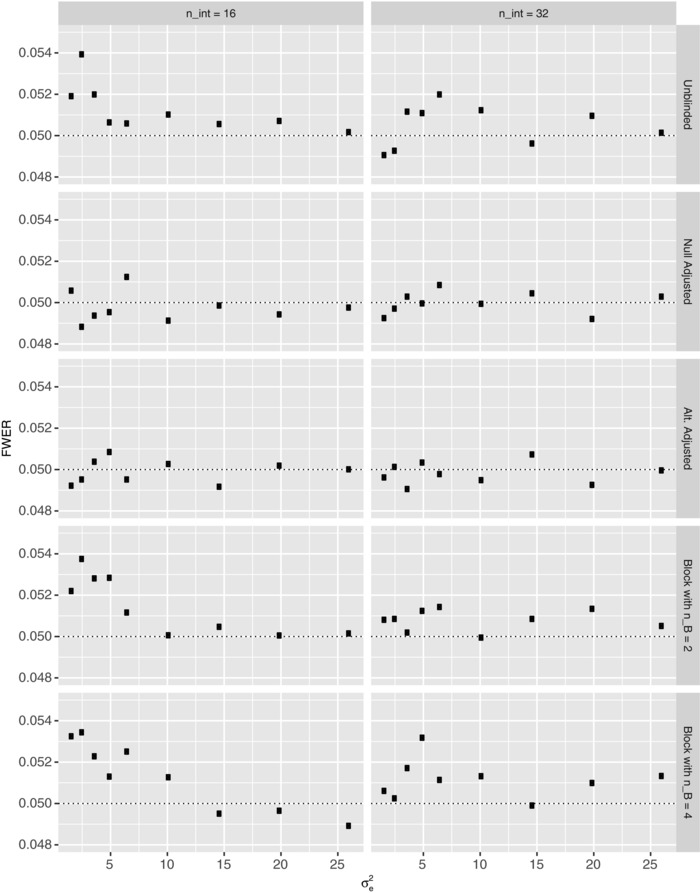
The simulated familywise error‐rate (FWER) is shown under the global null hypothesis for each of the reestimation procedures when $n_{\text{int}}\in \left\{16,32\right\}$, as a function of the within person variance $\sigma_{e}^{2}$, for Example 1. The Monte Carlo error is approximately 0.0007 in each instance. The dashed line indicates the desired value of the FWER

**Figure 4:**
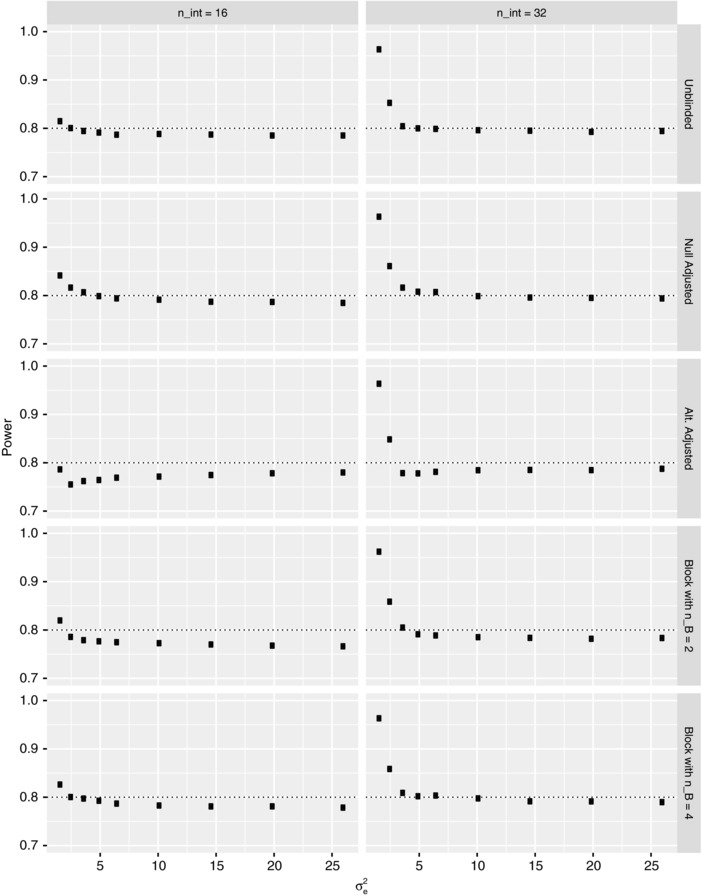
The simulated power is shown under the global alternative hypothesis for each of the reestimation procedures when $n_{\text{int}}\in \left\{16,32\right\}$, as a function of the within person variance $\sigma_{e}^{2}$, for Example 1. The Monte Carlo error is approximately 0.0013 in each instance. The dashed line indicates the desired value of the power

Figures 3 and 4 respectively present our results on the FWER and power of the various reestimation procedures when $n_{\text{int}}\in \left\{16,32\right\}$ for several values of $\sigma_{e}^{2}\in \left[0.25\left(6.51\right),4\left(6.51\right)\right]$, under the global null and alternative hypotheses, respectively. Corresponding findings for $\hat{N}$ are provided in the Supplementary Material.

Arguably, we observe that the FWER is more variable for smaller values of $\sigma_{e}^{2}$, with it changing little for several of the procedures when $\sigma_{e}^{2}>10$. There is additionally some evidence to suggest that increasing the value of $n_{\text{int}}$ reduces the overall effect $\sigma_{e}^{2}$ has on the FWER.

For the power, as would be anticipated, the reestimation procedures are over‐powered when $n_{\text{int}}=32$ and $\sigma_{e}^{2}$ is small. Moreover, increasing the value of $n_{\text{int}}$ universally increases the power. Finally, as $\sigma_{e}^{2}$ increases beyond approximately $\sigma_{e}^{2}=5$, for both considered values of $n_{\text{int}}$, there is little change in power.

### 3.6 Influence of δ

**Figure 5:**
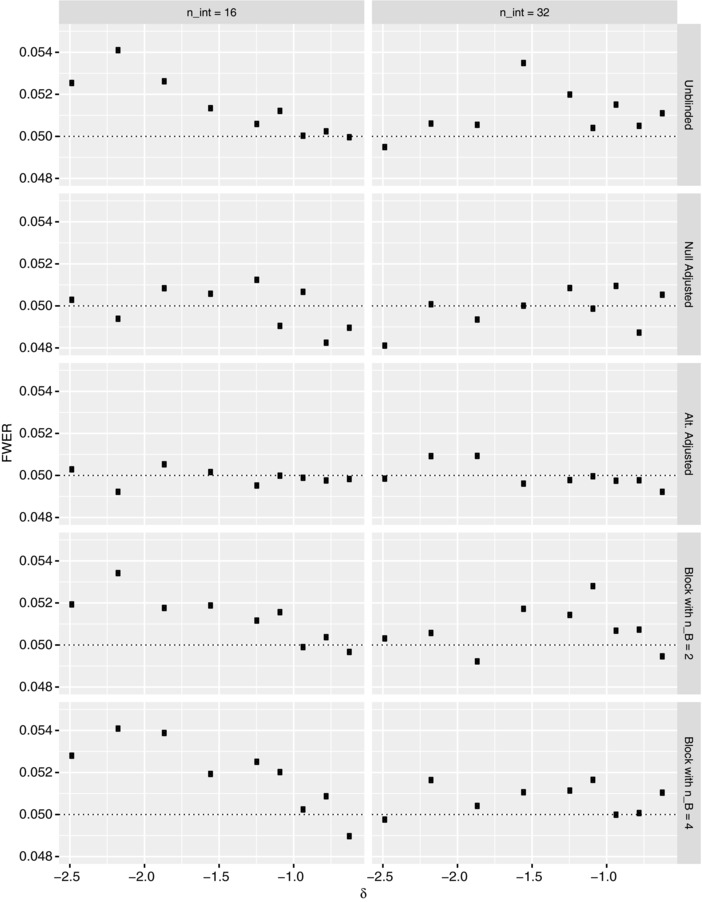
The simulated familywise error‐rate (FWER) is shown under the global null hypothesis for each of the reestimation procedures when $n_{\text{int}}\in \left\{16,32\right\}$, as a function of the clinically relevant difference δ, for Example 1. The Monte Carlo error is approximately 0.0007 in each instance. The dashed line indicates the desired value of the FWER

**Figure 6:**
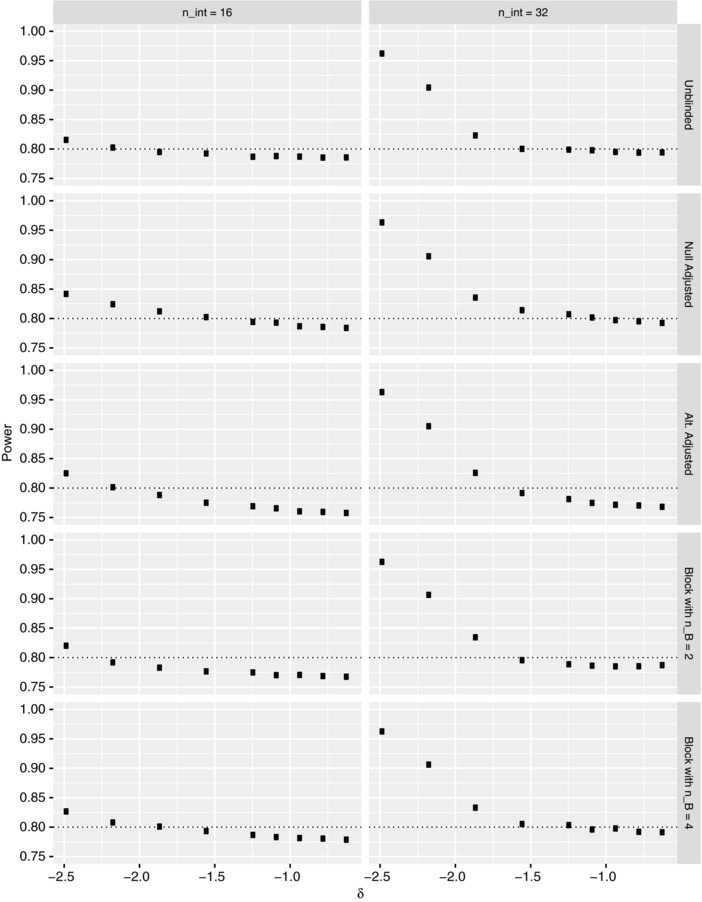
The simulated power is shown under the global alternative hypothesis for each of the reestimation procedures when $n_{\text{int}}\in \left\{16,32\right\}$, as a function of the clinically relevant difference δ, for Example 1. The Monte Carlo error is approximately 0.0013 in each instance. The dashed line indicates the desired value of the power

Here, we consider the case where $\pi_{2}=-0.77$, $\pi_{3}=-0.96$, $\pi_{4}=-0.55$, and $\sigma_{b}^{2}=10.12$, focusing on the influence δ has upon the procedures FWER and power. Precisely, Figures 5 and 6 respectively present our findings for the FWER and power of the various reestimation procedures when $n_{\text{int}}\in \left\{16,32\right\}$ for several values of δ∈[2(−1.24),0.5(−1.24)], under the global null and alternative hypotheses, respectively. Complimentary findings for $\hat{N}$ are provided in the Supplementary Material.

In Figure 5 we can see that there is no clear pattern to the effect on the FWER of changing δ, with the fluctuations for several of the estimators relatively small. However, there is some evidence to suggest that increasing the value of δ (i.e., making it closer to zero) reduces the FWER, as may be expected as this implies a larger requisite sample size.

Similar statements are true for the power when examining Figure 6. Analogous to our discussions around Figure 4, the reestimation procedures are over‐powered when $n_{\text{int}}=32$ and δ is large in magnitude. Furthermore, increasing the value of $n_{\text{int}}$ once more universally increases the power, while there appears to be a point beyond which the power remains relatively constant.

### 3.7 Sample size inflation factor

While the above suggests the overall performance of the reestimation is good, there are several simple refinements that can be implemented to improve the observed results.

One such refinement, to help ensure the power provided by the reestimation procedures is at least the desired 1−β, is to utilise a sample size inflation factor as originally proposed by Zucker et al. (1999). With it, the value of $\hat{N}$ as determined using the arguments above, is enlarged by the following factor

$${\left(\frac{t_{1-\alpha ,\nu_{n_{\text{int}}}}+t_{1-\beta ,\nu_{n_{\text{int}}}}}{z_{1-\alpha }+z_{1-\beta }}\right)}^{2}.$$

Of course, one must be careful that the new implied sample size does not exceed any specified value of $n_{\text{max}}$. However, this factor has then been shown to improve the performance of reestimation procedures in both superiority (Zucker et al., 1999), noninferiority (Friede & Kieser, 2013), and two‐treatment bioequivalence trials (Golkowski et al., 2014).

**Figure 7:**
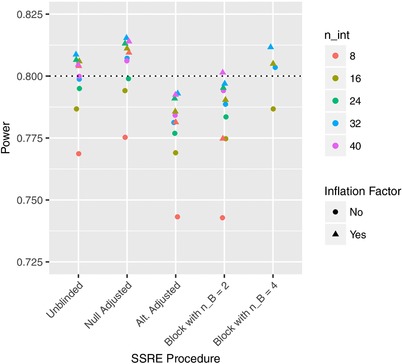
The influence of the considered inflation factor upon the power of the re‐estimation procedures under the global alternative hypothesis is shown for several values of $n_{\text{int}}$, for Example 1. The dashed line indicates the desired value of the power

Figure 7 displays its effect in the context of our multitreatment crossover trials. Explicitly, the power of the various reestimation procedures under the global alternative hypothesis, for $n_{\text{int}}\in \left\{8,16,24,32,40\right\}$ and $\sigma_{e}^{2}=6.51$, is shown with and without the use of the inflation factor. For the unblinded, null adjusted, and block randomized method with $n_{B}=4$, the inflation factor increases power to above the desired level in every instance. Consequently, this simple inflation factor appears once more to be an effective adjustment to the basic procedures.

## 4 DISCUSSION

In this article, we have developed and explored several methods for the interim re‐assessment of the sample size required by a multitreatment crossover trial. Our methodology is applicable to any trial analyzed using the linear‐mixed model (1), when there is equal participant allocation to a set of period‐balanced sequences. Thus while adapting the work of Golkowski et al. (2014) would be advisable in the case of an AB/BA superiority trial, given that it does not require the use of simulation, our methods are pertinent to a broader set of crossover designs. Indeed, they are as readily applicable to multitreatment superiority trials as they are ones for establishing bioequivalence.

We explored performance via three motivating examples, allowing consideration of settings with different types of sequences and a range of required sample sizes. Overall, the results presented here for the TOMADO trial are similar to those provided in the Supplementary Material for Examples 2 and 3. However, larger inflation to the FWER was observed in Example 2, most likely as a consequence of its associated smaller sample sizes. Nonetheless, the methods were found to provide desirable power characteristics with negligible inflation to the FWER in many settings. In particular, the blinded procedures provided comparable operating characteristics to the unblinded procedure, and thus can be considered viable alternatives. Following results for parallel arm trials (Friede & Kieser, 2013), the null adjusted blinded estimator arguably performed better than the other estimators in that its typical overestimation of the variance at interim led to the desired power being achieved more often. We may therefore tentatively suggest the null adjusted blinded estimator to be the preferred approach in this setting.

Our findings indicate that for each of the reestimation procedures, the choice of δ and the underlying values of $\sigma_{e}^{2}$ and $\sigma_{b}^{2}$ often have little effect upon the FWER and power. We may be reassured therefore that the performance of the procedures should often be relatively insensitive to the design parameters. On a similar note, it is important to recognize that one cannot be certain when utilizing these methods that the value of the period effects will not influence the performance of the reestimation procedures. While the final analysis should be asymptotically invariant to period effects, in finite samples it may influence the results of the hypothesis tests. Intuitively though one would not anticipate this effect to be large, nor would one routinely expect large period effects in many settings. In the Supplementary Material, simulations to explore this are presented for the TOMADO example. The results indicate that there is little evidence to suggest the value of the period effects influences the performance of the reestimation procedures. Trialists must be mindful however that this cannot be guaranteed, and should therefore be investigated.

We also considered the utility of a simple sample size inflation factor in ensuring the power reaches the desired level. Ultimately, we demonstrated that this was an effective extension to the basic reestimation procedures. Though the observed inflation to the FWER of our procedures was often small, if more strict control is desired, a crude α‐level adjustment procedure can also be utilized. For a particular reestimation scenario, the values of $\sigma_{e}^{2}$ and $\sigma_{b}^{2}$, $\sigma_{e,\text{max}}^{2}$ , and $\sigma_{b,\text{max}}^{2}$ say, which maximize the inflation to the FWER under the global null hypothesis can be determined via a two dimensional search. Then, the significance level used in the analysis of the trial can be adjusted to the $\alpha_{\text{adj}}$ that confers a FWER of α for this $\sigma_{e,\text{max}}^{2}$, $\sigma_{b,\text{max}}^{2}$ pair, according to further simulations. This may be useful in practice if the inflation is large for a particular trial design scenario of interest.

It is important to note the seemingly inherent advantages and disadvantages of the various reestimation procedures. The adjusted estimator is perhaps the most constrained of those considered; requiring an equal number of patients to be allocated to each sequence for any nonzero adjustment to be reasonable. This is particularly troubling because of the possibility of patient drop‐out.

The estimator following block randomisation does not necessitate equal allocation to sequences (though its performance was considered here only when this was the case), but could also fall foul of patient drop‐out that would prevent the estimation of the within person variance for each block. It also requires block randomization, and could not be used with a more simple randomization scheme if this was desired. The unblinded estimator of course suffers from none of these problems, but as discussed may be looked upon less favorably by regulators.

Finally, note that in conducting our work we also considered the performance of two reestimation procedures based on methodology for the clustering of longitudinal data (Fraley & Raftery, 2003; Genolini, Alacoque, Sentenac, & Arnauld, 2009). The motivation for this came from the Expectation‐Maximisation algorithm approaches of Gould and Shih (1992) for parallel two‐arm, and Kieser and Friede (2002) for parallel multiarm, studies. These methods may seem appealing, as they are blinded, under certain assumptions can produce unbiased estimates of the variance parameters, do not require specification of any adjustment, and in theory should be able to more readily handle small amounts of missing data. However, we found that they routinely vastly underestimated the size of within person variance, resulting in substantially lower power than that attained by the other reestimation procedures. Accordingly, especially given the associated concerns about the appropriateness of an Expectation‐Maximization algorithm for blinded sample size reestimation (Friede & Kieser, 2002), we would not recommend reestimation be performed based on a clustering‐based approach.

In conclusion, following findings for other trial design settings, blinded estimators can be used for sample size reestimation in multitreatment crossover trials. The operating characteristics of any chosen procedure should of course be assessed pretrial through a comprehensive simulation study. But, often, investigators can hope to find that the likelihood of correctly powering their study when there is pretrial uncertainty over the within and between person variances can be enhanced.

## CONFLICT OF INTEREST

The authors have declared no conflict of interest.

## Supporting information

Supporting Information

Supporting Information
