# Supplementary material for: Blinded and unblinded sample size reestimation in crossover trials balanced for period
Source: Biom J. 2018 Aug 3;60(5):917–33. doi: 10.1002/bimj.201700092 (PMC6175184; doi:10.1002/bimj.201700092)
Supplement: Supplementary file 1 — Supporting Information [file BIMJ-60-917-s001.pdf]

Supplementary Material to "Blinded and unblinded sample size re-estimation in crossover trials balanced for period"

S.M.1. Deriving $\mathrm{N}(\sigma_e^2, \sigma_b^2)$

In this section, we elaborate on how a formulae for the sample size required by a trial, $\mathrm{N}(\sigma_e^2, \sigma_b^2)$, can be specified when σ_e^2 and σ_b^2 are known, a set of K sequences have been chosen, and $n_1 = \cdots = n_K$. First, we focus on the set up from Section 2.1, before briefly describing adjustments for other testing scenarios.

We begin by denoting the linear mixed model for our vector of observations $\boldsymbol{y}$ by $\boldsymbol{y} = \boldsymbol{X\beta} + \boldsymbol{Zb} + \boldsymbol{\epsilon}$. Here, it is important to note that the particular form of the matrices $\boldsymbol{X}$ and $\boldsymbol{Z}$ is dependent on the sample size and the chosen sequences. Then, the generalised least squares estimate for $\boldsymbol{\beta}$, $\hat{\boldsymbol{\beta}}$, is given by

$$\hat{\boldsymbol{\beta}} = (\boldsymbol{X}^\top \boldsymbol{\Sigma}^{-1} \boldsymbol{X})^{-1} \boldsymbol{X}^\top \boldsymbol{\Sigma}^{-1} \boldsymbol{y},$$

where $\boldsymbol{\Sigma} = \boldsymbol{ZGZ}^\top + \boldsymbol{R}$ is known. Precisely, by our choice of covariance structure implied by linear mixed model (1), $\boldsymbol{\Sigma}$ will be an $NP \times NP$ block diagonal matrix, consisting of $P \times P$ blocks given by $\sigma_e^2 \boldsymbol{I}_P + \sigma_b^2 \boldsymbol{J}_P$. In addition, $\mathrm{Var}(\hat{\boldsymbol{\beta}}) = (\boldsymbol{X}^\top \boldsymbol{\Sigma}^{-1} \boldsymbol{X})^{-1}$, will also be known. Finally, $\hat{\boldsymbol{\beta}}$ is an unbiased estimate of $\boldsymbol{\beta}$. Thus the vector of test statistics $\boldsymbol{Q} = (Q_1, \dots, Q_{D-1})^\top$, where

$$Q_d = \frac{\hat{\tau}_d}{\{\mathrm{Var}(\hat{\tau}_d)\}^{1/2}}, \qquad d = 1, \dots, D-1,$$

has the following multivariate normal distribution

$$\boldsymbol{Q} \sim N\left\{\boldsymbol{\tau} \circ \boldsymbol{I}^{1/2}, \boldsymbol{Diag}(\boldsymbol{I}^{1/2})\mathrm{Var}(\hat{\boldsymbol{\tau}})\boldsymbol{Diag}(\boldsymbol{I}^{1/2})\right\}.$$

Here, $\boldsymbol{\tau} = (\tau_1, \dots, \tau_{D-1})^\top$, and $\boldsymbol{I} = (I_1, \dots, I_{D-1})^\top$ with $I_d = \{\mathrm{Var}(\hat{\tau}_d)\}^{-1}$. Furthermore, $\boldsymbol{Diag}(\boldsymbol{v})$ is the matrix formed by placing the elements of the vector $\boldsymbol{v}$ along the leading diagonal, and we take the convention that $\{(v_1, \dots, v_m)^\top\}^{1/2} = (v_1^{1/2}, \dots, v_m^{1/2})^\top$.

Using the distribution of $\boldsymbol{Q}$, we can control the FWER to a desired level α for our hypotheses of interest by rejecting H_{0d} if $Q_d > e$, for the e which is the solution of

$$1 - \alpha = \Phi_{D-1}\{(e, \dots, e)^\top, \mathrm{Var}(\boldsymbol{Q})\},$$

where $\Phi_M\{\mathbf{x}, \Lambda\}$ is the M-dimensional cumulative distribution function of a central multivariate normal distribution with covariance matrix Λ. The power to reject H_{01} when $\tau_1 = \delta$ is then

$$\Phi_1\{\delta I_1^{1/2} - \Phi_1^{-1}(1 - \alpha_*, 1), 1\},$$

for $\alpha_* = 1 - \Phi_1(e, 1)$. Consequently, to have power of $1 - \beta$ we must have

$$\Phi_1\{\delta I_1^{1/2} - \Phi_1^{-1}(1 - \alpha_*, 1), 1\} = 1 - \beta.$$

By deriving the explicit form of I_1 we can determine its dependence upon the sample size N, which allows the above to be arranged and our final formula $\mathrm{N}(\sigma_e^2, \sigma_b^2)$ specified.

For example, as discussed in Section 3.2, in the case where complete block sequences that are balanced for period are utilised, it is well known that $I_1 = N/(2\sigma_e^2)$ (Jones and Kenward, 2014). Thus, we can rearrange the above to acquire our previously specified formula

$$\mathrm{N}(\sigma_e^2, \sigma_b^2) \equiv \mathrm{N}(\sigma_e^2) = \frac{2\sigma_e^2\{\Phi_1^{-1}(1 - \alpha_*, 1) + \Phi_1^{-1}(1 - \beta, 1)\}^2}{\delta^2} \equiv \frac{2\sigma_e^2(z_{1-\alpha_*} + z_{1-\beta})^2}{\delta^2}.$$

In the case where alternative hypotheses are to be assessed (e.g., a global test that foregoes many-to-one comparisons), provided testing is performed using effects from $\hat{\boldsymbol{\beta}}$, the above can easily be adapted to designate an appropriate sample size formula. In each instance, one specifies a vector of test statistics, the distribution of which can be derived using that of $\hat{\boldsymbol{\beta}}$. This allows an appropriate formulae for controlling the FWER to be provided. For example, in the case where

$$H_{0d} : \tau_d = 0, \qquad H_{1d} : \tau_d \neq 0, \qquad d = 1, \dots, D - 1,$$

we still utilise $\boldsymbol{Q}$ as defined above, but now reject H_{0d} if $|Q_d| > e$, where e is the solution of

$$1 - \alpha/2 = \Phi_{D-1}\{(e, \dots, e)^\top, \mathrm{Var}(\boldsymbol{Q})\}.$$

Then, a formulae for the power of interest can similarly be designated, which allows N to either be specified explicitly, or permits an iterative search to be performed to determine its required value.

For example, for the design scenario of Section 2.1, if we instead desire a familywise power of at least $1 - \beta$ when $\boldsymbol{\tau} = (\delta, \dots, \delta)^\top$ (that is, power to reject at least one of $H_{01}, \dots, H_{0D-1}$), our formula for the power becomes

$$1 - \Phi_{D-1}\{(e - \delta I_1^{1/2}, \dots, e - \delta I_{D-1}^{1/2})^\top, \mathrm{Var}(\boldsymbol{Q})\}.$$

Having derived $\boldsymbol{I}$ and $\mathrm{Var}(\boldsymbol{Q})$ for any N, a one-dimensional search can then be performed to acquire the minimal N such that the above is at least $1 - \beta$.

S.M.2. Adjusted blinded estimator

In this section, we find the expected value of the adjusted blinded estimators of the within and between person variances discussed in the main paper.

First, note that for any K sequences which are balanced for period

$$\sum_{k=1}^{K}(\tau_{\mathrm{d}(j,k)} - \tau_{\mathrm{d}(j-1,k)}) = 0, \tag{3}$$

for $j = 2, \dots, P$, and

$$\sum_{k=1}^{K}\tau_{\mathrm{d}(1,k)} = \cdots = \sum_{k=1}^{K}\tau_{\mathrm{d}(P,k)}. \tag{4}$$

Now note that for the linear mixed model (1) that

$$\mathrm{E}(p_{ijk}) = \pi_j - \pi_{j-1} + \tau_{\mathrm{d}(j,k)} - \tau_{\mathrm{d}(j-1,k)}.$$

In addition, observe that $\mathrm{Cov}(p_{i_1 j_1 k_1}, p_{i_2 j_2 k_2}) = 0$ unless $i_1 = i_2$ and $k_1 = k_2$. Furthermore

$$\begin{aligned}
\mathrm{Var}(p_{ijk}) &= \mathrm{Cov}(y_{ijk} - y_{ij-1k}, y_{ijk} - y_{ij-1k}),\\
&= \mathrm{Cov}(y_{ijk}, y_{ijk}) - \mathrm{Cov}(y_{ijk}, y_{ij-1k})\\
&\quad - \mathrm{Cov}(y_{ij-1k}, y_{ijk}) + \mathrm{Cov}(y_{ij-1k}, y_{ij-1k}),\\
&= (\sigma_e^2 + \sigma_b^2) - \sigma_b^2 - \sigma_b^2 + (\sigma_e^2 + \sigma_b^2),\\
&= 2\sigma_e^2.
\end{aligned}$$

Consequently

$$\begin{aligned}
\mathrm{E}\left(\bar{p}_j\right) &= \mathrm{E}\left(\frac{1}{n_{\mathrm{int}}}\sum_{k=1}^{K}\sum_{i=1}^{n_{\mathrm{int}}/K} p_{ijk}\right),\\
&= \frac{1}{n_{\mathrm{int}}}\sum_{k=1}^{K}\sum_{i=1}^{n_{\mathrm{int}}/K} \mathrm{E}(p_{ijk}),\\
&= \frac{1}{n_{\mathrm{int}}}\sum_{k=1}^{K}\sum_{i=1}^{n_{\mathrm{int}}/K} (\pi_j - \pi_{j-1} + \tau_{\mathrm{d}(j,k)} - \tau_{\mathrm{d}(j-1,k)}),\\
&= \frac{1}{n_{\mathrm{int}}} n_{\mathrm{int}}(\pi_j - \pi_{j-1}),\\
&= \pi_j - \pi_{j-1},
\end{aligned}$$

where we have used Equation (3). Additionally

$$\begin{aligned}
\mathrm{Var}\left(\bar{p}_j\right) &= \mathrm{Var}\left(\frac{1}{n_{\mathrm{int}}}\sum_{k=1}^{K}\sum_{i=1}^{n_{\mathrm{int}}/K} p_{ijk}\right),\\
&= \frac{1}{n_{\mathrm{int}}^2}\mathrm{Var}\left(\sum_{k=1}^{K}\sum_{i=1}^{n_{\mathrm{int}}/K} p_{ijk}\right),\\
&= \frac{1}{n_{\mathrm{int}}^2}\left\{\sum_{k=1}^{K}\sum_{i=1}^{n_{\mathrm{int}}/K} \mathrm{Var}\left(p_{ijk}\right) + \sum_{k_1\neq k_2}\sum_{i_1\neq i_2} \mathrm{Cov}\left(p_{i_1 j k_1}, p_{i_2 j k_2}\right)\right\},\\
&= \frac{1}{n_{\mathrm{int}}^2}\left\{\sum_{k=1}^{K}\sum_{i=1}^{n_{\mathrm{int}}/K} \mathrm{Var}\left(p_{ijk}\right)\right\},\\
&= \frac{1}{n_{\mathrm{int}}^2}(2\sigma_e^2 n_{\mathrm{int}}),\\
&= \frac{2\sigma_e^2}{n_{\mathrm{int}}}.
\end{aligned}$$

Next, consider the following, which we call $\sigma^2_{\mathrm{within}}$

$$\begin{aligned}
\sigma^2_{\mathrm{within}} &= \frac{1}{2(P-1)(n_{\mathrm{int}}-1)}\sum_{j=2}^{P}\sum_{k=1}^{K}\sum_{i=1}^{n_{\mathrm{int}}/K}(p_{ijk} - \bar{p}_j)^2,\\
&= \frac{1}{2(P-1)(n_{\mathrm{int}}-1)}\sum_{j=2}^{P}\left(\sum_{k=1}^{K}\sum_{i=1}^{n_{\mathrm{int}}/K} p_{ijk}^2 - n_{\mathrm{int}}\bar{p}_j^2\right).
\end{aligned}$$

Taking expectations we have

$$\begin{aligned}
2(P-1)(n_{\text{int}}-1)\mathrm{E}(\sigma^2_{\text{within}}) &= \sum_{j=2}^{P}\left\{\sum_{k=1}^{K}\sum_{i=1}^{n_{\text{int}}/K}\mathrm{E}\left(p_{ijk}^2\right) - n_{\text{int}}\mathrm{E}\left(\bar{p}_j^2\right)\right\},\\
&= \sum_{j=2}^{P}\Bigg[\sum_{k=1}^{K}\sum_{i=1}^{n_{\text{int}}/K}\left\{\mathrm{Var}(p_{ijk}) + \mathrm{E}(p_{ijk})^2\right\}\\
&\qquad - n_{\text{int}}\left\{\mathrm{Var}(\bar{p}_j) + \mathrm{E}(\bar{p}_j)^2\right\}\Bigg],\\
&= \sum_{j=2}^{P}\Bigg[\sum_{k=1}^{K}\sum_{i=1}^{n_{\text{int}}/K}\left\{2\sigma_e^2 + (\pi_j - \pi_{j-1} + \tau_{\mathrm{d}(j,k)} - \tau_{\mathrm{d}(j-1,k)})^2\right\}\\
&\qquad -n_{\text{int}}\left\{\frac{2\sigma_e^2}{n_{\text{int}}} + (\pi_j - \pi_{j-1})^2\right\}\Bigg],\\
&= \sum_{j=2}^{P}\Bigg\{2(n_{\text{int}}-1)\sigma_e^2 + \sum_{k=1}^{K}\sum_{i=1}^{n_{\text{int}}/K}(\pi_j - \pi_{j-1} + \tau_{\mathrm{d}(j,k)} - \tau_{\mathrm{d}(j-1,k)})^2\\
&\qquad - n_{\text{int}}(\pi_j - \pi_{j-1})^2\Bigg\},\\
&= 2(P-1)(n_{\text{int}}-1)\sigma_e^2\\
&\quad + \sum_{j=2}^{P}\Bigg\{\sum_{k=1}^{K}\sum_{i=1}^{n_{\text{int}}/K}(\pi_j - \pi_{j-1})^2 + \sum_{k=1}^{K}\sum_{i=1}^{n_{\text{int}}/K}(\tau_{\mathrm{d}(j,k)} - \tau_{\mathrm{d}(j-1,k)})^2\\
&\qquad + 2(\pi_j - \pi_{j-1})\sum_{k=1}^{K}\sum_{i=1}^{n_{\text{int}}/K}(\tau_{\mathrm{d}(j,k)} - \tau_{\mathrm{d}(j-1,k)})\\
&\qquad - n_{\text{int}}(\pi_j - \pi_{j-1})^2\Bigg\},\\
&= 2(P-1)(n_{\text{int}}-1)\sigma_e^2\\
&\quad + \sum_{j=2}^{P}\Bigg\{n_{\text{int}}(\pi_j - \pi_{j-1})^2 + \sum_{k=1}^{K}\sum_{i=1}^{n_{\text{int}}/K}(\tau_{\mathrm{d}(j,k)} - \tau_{\mathrm{d}(j-1,k)})^2\\
&\qquad - n_{\text{int}}(\pi_j - \pi_{j-1})^2\Bigg\},\\
&= 2(P-1)(n_{\text{int}}-1)\sigma_e^2 + \sum_{j=2}^{P}\sum_{k=1}^{K}\sum_{i=1}^{n_{\text{int}}/K}(\tau_{\mathrm{d}(j,k)} - \tau_{\mathrm{d}(j-1,k)})^2,\\
&= 2(P-1)(n_{\text{int}}-1)\sigma_e^2 + \frac{n_{\text{int}}}{K}\sum_{j=2}^{P}\sum_{k=1}^{K}(\tau_{\mathrm{d}(j,k)} - \tau_{\mathrm{d}(j-1,k)})^2.
\end{aligned}$$

Thus we have that

$$\mathrm{E}(\sigma^2_{\text{within}}) = \sigma_e^2 + \frac{n_{\text{int}}}{2K(P-1)(n_{\text{int}}-1)}\sum_{j=2}^{P}\sum_{k=1}^{K}(\tau_{\mathrm{d}(j,k)} - \tau_{\mathrm{d}(j-1,k)})^2,$$

and for our adjusted blinded estimator

$$\hat{\sigma}_e^2 = \sigma^2_{\text{within}} - \frac{n_{\text{int}}}{2K(P-1)(n_{\text{int}}-1)} \sum_{j=2}^{P}\sum_{k=1}^{K}(\tau^*_{\text{d}(j,k)} - \tau^*_{\text{d}(j-1,k)})^2,$$

it is clear that if $\tau_d^* = \tau_d$ for $d = 1, \dots, D-1$ then $\text{E}(\hat{\sigma}_e^2) = \sigma_e^2$, and $\hat{\sigma}_e^2$ is an unbiased estimator for σ_e^2 as claimed.

For the case where the sequences utilised for treatment allocation are additionally complete-block, the above estimator for σ_e^2 is all that is required by the adjusted blinded re-estimation procedures. When this is not the case however, we further require a blinded estimate of the between person variance σ_b^2. For this, define

$$q_{ijk} = y_{ij-1k} + y_{ijk},$$
$$\bar{q}_j = \frac{1}{n_{\text{int}}}\sum_{k=1}^{K}\sum_{i=1}^{n_{\text{int}}/K} q_{ijk}.$$

Then, by a direct adaptation of the arguments above, and by using Equation (4), we can show that

$$\text{E}(q_{ijk}) = \pi_{j-1} + \pi_j + \tau_{\text{d}(j-1,k)} + \tau_{\text{d}(j,k)},$$
$$\text{Var}(q_{ijk}) = 2(\sigma_e^2 + 2\sigma_b^2),$$
$$\text{E}(\bar{q}_j) = \pi_{j-1} + \pi_j + \frac{2}{D}\sum_{k=1}^{K}\tau_{\text{d}(1,k)},$$
$$\text{Var}(\bar{q}_j) = \frac{2(\sigma_e^2 + 2\sigma_b^2)}{n_{\text{int}}}.$$

Now define

$$\sigma^2_{\text{between}} = \frac{1}{2(P-1)(n_{\text{int}}-1)}\sum_{j=2}^{P}\sum_{k=1}^{K}\sum_{i=1}^{n_{\text{int}}/K}(q_{ijk} - \bar{q}_j)^2.$$

Modifying the derivations for σ^2_{within}, we have that

$$\text{E}(\sigma^2_{\text{between}}) = \sigma_e^2 + 2\sigma_b^2 + \frac{n_{\text{int}}}{2K(P-1)(n_{\text{int}}-1)}\sum_{j=2}^{P}\sum_{k=1}^{K}(\tau_{\text{d}(j-1,k)} + \tau_{\text{d}(j,k)})^2 + \frac{2n_{\text{int}}}{D^2(n_{\text{int}}-1)}\left(\sum_{k=1}^{K}\tau_{\text{d}(1,k)}\right)^2.$$

We can thus then take our blinded estimate for σ_b^2, $\hat{\sigma}_b^2$, as

$$\hat{\sigma}_b^2 = \frac{1}{2}\left\{\sigma^2_{\text{between}} - \hat{\sigma}_e^2 - \frac{n_{\text{int}}}{2K(P-1)(n_{\text{int}}-1)}\sum_{j=2}^{P}\sum_{k=1}^{K}(\tau^*_{\text{d}(j-1,k)} + \tau^*_{\text{d}(j,k)})^2 - \frac{2n_{\text{int}}}{D^2(n_{\text{int}}-1)}\left(\sum_{k=1}^{K}\tau^*_{\text{d}(1,k)}\right)^2\right\}.$$

Again, in the case that $\tau_d^* = \tau_d$ for $d = 1, \dots, D-1$, this is an unbiased estimator for σ_b^2.

S.M.3. Blinded estimator following block randomisation

Here, we first demonstrate the forwarded blinded estimator for σ_e^2 following block randomisation is also unbiased. Throughout, the index k above is essentially replaced by the index b. To begin, observe that

$$\mathrm{E}(p_{ijb}) = \pi_j - \pi_{j-1} + \tau_{\mathrm{d}_B(j,b)} - \tau_{\mathrm{d}_B(j-1,b)},$$

where $\mathrm{d}_B(j,b)$ is the index of the treatment given to a patient in block b in period j. As above, $\mathrm{Cov}(p_{i_1 j_1 b_1}, p_{i_2 j_2 b_2}) = 0$ unless $i_1 = i_2$ and $b_1 = b_2$, and $\mathrm{Cov}(p_{ijb}, p_{ijb}) = 2\sigma_e^2$. Consequently

$$\begin{aligned}
\mathrm{E}\left(\bar{p}_{jb}\right) &= \mathrm{E}\left(\frac{1}{n_B}\sum_{i=1}^{n_B} p_{ijb}\right), \\
&= \frac{1}{n_B}\sum_{i=1}^{n_B}\mathrm{E}(p_{ijb}), \\
&= \frac{1}{n_B}\sum_{i=1}^{n_B}(\pi_j - \pi_{j-1} + \tau_{\mathrm{d}_B(j,b)} - \tau_{\mathrm{d}_B(j-1,b)}), \\
&= \frac{1}{n_B} n_B(\pi_j - \pi_{j-1} + \tau_{\mathrm{d}_B(j,b)} - \tau_{\mathrm{d}_B(j-1,b)}), \\
&= \pi_j - \pi_{j-1} + \tau_{\mathrm{d}_B(j,b)} - \tau_{\mathrm{d}_B(j-1,b)}, \\
\mathrm{Var}\left(\bar{p}_{jb}\right) &= \mathrm{Var}\left(\frac{1}{n_B}\sum_{i=1}^{n_B} p_{ijb}\right), \\
&= \frac{1}{n_B^2}\mathrm{Var}\left(\sum_{i=1}^{n_B} p_{ijb}\right), \\
&= \frac{1}{n_B^2}\left\{\sum_{i=1}^{n_B}\mathrm{Var}\left(p_{ijb}\right) + \sum_{i_1 \neq i_2}\mathrm{Cov}\left(p_{i_1 jb}, p_{i_2 jb}\right)\right\}, \\
&= \frac{1}{n_B^2}\left\{\sum_{i=1}^{n_B}\mathrm{Var}\left(p_{ijb}\right)\right\}, \\
&= \frac{1}{n_B^2}(2\sigma_e^2 n_B), \\
&= \frac{2\sigma_e^2}{n_B}.
\end{aligned}$$

Next consider the proposed blinded estimator

$$\begin{aligned}
\hat{\sigma}_e^2 &= \frac{1}{2(P-1)(n_{\mathrm{int}} - B)}\sum_{j=2}^{P}\sum_{b=1}^{B}\sum_{i=1}^{n_B}(p_{ijb} - \bar{p}_{jb})^2, \\
&= \frac{1}{2(P-1)(n_{\mathrm{int}} - B)}\sum_{j=2}^{P}\left[\sum_{b=1}^{B}\left(\sum_{i=1}^{n_B} p_{ijb}^2 - n_B\bar{p}_{jb}^2\right)\right].
\end{aligned}$$

We have

$$2(P-1)(n_{\mathrm{int}} - B)\mathrm{E}(\hat{\sigma}_e^2) = \sum_{j=2}^{P}\left\{\sum_{b=1}^{B}\sum_{i=1}^{n_B}\mathrm{E}\left(p_{ijb}^2\right) - n_B\sum_{b=1}^{B}\mathrm{E}\left(\bar{p}_{jb}^2\right)\right\},$$

$$
\begin{aligned}
&= \sum_{j=2}^{P} \left[\sum_{b=1}^{B}\sum_{i=1}^{n_B} \left\{ \mathrm{Var}(p_{ijb}) + \mathrm{E}(p_{ijb})^2 \right\} \right. \\
&\qquad \left. - n_B \sum_{b=1}^{B} \left\{ \mathrm{Var}(\bar{p}_{jb}) + \mathrm{E}(\bar{p}_{jb})^2 \right\} \right], \\
&= \sum_{j=2}^{P} \left[\sum_{b=1}^{B}\sum_{i=1}^{n_B} \left\{ 2\sigma_e^2 + (\pi_j - \pi_{j-1} + \tau_{\mathrm{d}_B(j,b)} - \tau_{\mathrm{d}_B(j-1,b)})^2 \right\} \right. \\
&\qquad \left. - n_B \sum_{b=1}^{B} \left\{ \frac{2\sigma_e^2}{n_B} + (\pi_j - \pi_{j-1} + \tau_{\mathrm{d}_B(j,b)} - \tau_{\mathrm{d}_B(j-1,b)})^2 \right\} \right], \\
&= \sum_{j=2}^{P} \left[2(n_{\mathrm{int}} - B)\sigma_e^2 + \sum_{b=1}^{B}\sum_{i=1}^{n_B} (\pi_j - \pi_{j-1} + \tau_{\mathrm{d}_B(j,b)} - \tau_{\mathrm{d}_B(j-1,b)})^2 \right. \\
&\qquad \left. - n_B \sum_{b=1}^{B} (\pi_j - \pi_{j-1} + \tau_{\mathrm{d}_B(j,b)} - \tau_{\mathrm{d}_B(j-1,b)})^2 \right], \\
&= \sum_{j=2}^{P} \left[2(n_{\mathrm{int}} - B)\sigma_e^2 \right], \\
&= 2(P-1)(n_{\mathrm{int}} - B)\sigma_e^2.
\end{aligned}
$$

Thus $\mathrm{E}(\hat{\sigma}_e^2) = \sigma_e^2$, and the estimator is unbiased.

For non-complete-block treatment allocation, we can in this case take

$$
\hat{\sigma}_b^2 = \frac{1}{2} \left\{ \frac{1}{2(P-1)(n_{\mathrm{int}} - B)} \sum_{j=2}^{P}\sum_{b=1}^{B}\sum_{i=1}^{n_B} (q_{ijb} - \bar{q}_{jb})^2 - \hat{\sigma}_e^2 \right\}.
$$

To show this is unbiased, again without loss of generality consider the case where $\mu_0 = 0$. Then

$$
\begin{aligned}
\mathrm{E}(q_{ijb}) &= \pi_j + \pi_{j-1} + \tau_{\mathrm{d}_B(j,b)} + \tau_{\mathrm{d}_B(j-1,b)}, \\
\mathrm{Var}(q_{ijb}) &= 2(\sigma_e^2 + 2\sigma_b^2), \\
\mathrm{E}(\bar{q}_{jb}) &= \pi_j + \pi_{j-1} + \tau_{\mathrm{d}_B(j,b)} + \tau_{\mathrm{d}_B(j-1,b)}, \\
\mathrm{Var}(\bar{q}_{jb}) &= \frac{2(\sigma_e^2 + 2\sigma_b^2)}{n_B}.
\end{aligned}
$$

Using these results, a direct modification of the derivation for $\hat{\sigma}_e^2$ gives $\mathrm{E}(\hat{\sigma}_b^2) = \sigma_b^2$.

S.M.4. Example 1: TOMADO

S.M.4.1. Introduction

In this section, we expand on our results from Section 3 on Example 1; the TOMADO trial. Throughout, we set

$$
\mu_0 = 10.65,\ \sigma_e^2 = 6.51,\ \alpha = 0.05,\ \beta = 0.2.
$$

However, we now consider, in turn, the effect of altering the values of σ_b^2, δ, and the π_j, from those used in Section 3.

S.M.4.2. Influence of σ_e^2

First, we provide additional results corresponding to Section 3.5. Specifically, in Supplementary Figures 1 and 2 present the distributions of the $\hat{N}$ for the various re-estimation procedures when $n_{\text{int}} \in \{16, 32\}$ for several values of $\sigma_e^2 \in [0.25(6.51), 4(6.51)]$, under the global null and alternative hypotheses respectively. Our results are as we would anticipate; that when σ_e^2 is small the trials tend to be over-powered, and thus $\hat{N} \approx n_{\text{int}}$ in most instances. However, for large σ_e^2 there is more substantial variation in the value of $\hat{N}$.

S.M.4.3. Influence of σ_b^2

We now examine, in the case where $\pi_2 = -0.77$, $\pi_3 = -0.96$, $\pi_4 = -0.55$, and $\delta = -1.24$, the influence of the value of σ_b^2 on the performance of the re-estimation procedures. As in Section 3.5, we would like to ascertain the effect changing σ_b^2 has upon the FWER and power.

Supplementary Figures 3 and 4 respectively present our results on the FWER and power of the various re-estimation procedures when $n_{\text{int}} \in \{16, 32\}$ for several values of $\sigma_b^2 \in [0.25(10.12), 4(10.12)]$, under the global null and alternative hypotheses respectively.

As would be anticipated, the value of σ_b^2 appears to have extremely little effect upon the performance of each of the re-estimation procedures. Recall that this is a consequence of the fact that the complete block sequences utilised in Example 1 render the requisite sample size independent of the between person variance.

S.M.4.4. Influence of δ

Next, we provide additional results corresponding to Section 3.6. Explicitly, in Supplementary Figures 5 and 6 present the distributions of the $\hat{N}$ for the various re-estimation procedures when $n_{\text{int}} \in \{16, 32\}$ for several values of $\delta \in [2(-1.24), 0.5(-1.24)]$, under the global null and alternative hypotheses respectively. As in Section S.M.4.2, our results are as we would expect. When δ is large the trials are typically determined to be over-powered, and thus generally $\hat{N} \approx n_{\text{int}}$. However, for small δ there is larger variation in the values of $\hat{N}$.

S.M.4.5. Influence of period effects

In Section 4, we discussed how the influence of the period effects should be examined using simulation. Again, though the final analysis on the trials data is asymptotically invariant to the true value of the period effects, their value may influence the ability of the various re-estimation procedures to accurately estimate the variance parameters at the interim reassessment. In this section, we assess the influence of the value of the period effects on each of the re-estimation procedures. Explicitly, we consider the case with $n_{\text{int}} = 16$ and $\sigma_e^2 = 6.51$, under the global null hypothesis. All others parameters are left as specified in Section 3.2, except for the π_j for $j = 2, 3, 4$. For these, in each replicate simulation we take

$$\pi_j \sim N(\hat{\pi}_j, \sigma_\pi^2), \qquad j = 2, 3, 4.$$

That is, a value for each π_j is drawn from a normal distribution with mean $\hat{\pi}_j$, and specified variance σ_π^2. We then assess the effect of the size of σ_π^2 upon the trials FWER and values for $\hat{\sigma}_e^2$. Supplementary Figures 7 and 8 display our findings. From them, it does appear that at least for this design scenario and associated parameter set, the value of the period effects tends to have little influence on the interim estimation of the within person variance, or on the trials FWER.

S.M.5. Example 2: Formoterol

S.M.5.1. Introduction

Senn (2002) reported the results of a double-blind placebo controlled cross-over trial to assess the performance of two doses of formoterol solution aerosol ($D = 3$). Denoting the treatments by $d = 0, 1, 2$, with $d = 0$ the placebo and $d = 1, 2$ corresponding to the two doses of formoterol, patients were allocated to one of the following six incomplete block sequences

$$01,\ 10,\ 02,\ 20,\ 12,\ 21.$$

We assume that the primary FEV1 (forced expiratory volume in 1 second) outcome data was to be analysed with the linear mixed model (1), in order to test the following hypotheses

$$H_{0d} : \tau_d \leq 0, \qquad H_{1d} : \tau_d > 0, \qquad d = 1, 2.$$

A complete case analysis of the data presented by Senn (2002) gives the following estimates for the parameters in the model

$$\hat{\mu}_0 = 1.51,\ \hat{\pi}_2 = 0.03,\ \hat{\tau}_1 = 0.50,\ \hat{\tau}_2 = 0.52,\ \hat{\sigma}_e^2 = 0.053,\ \hat{\sigma}_b^2 = 0.49.$$

Consequently, for $\alpha = 0.1$ and $\beta = 0.2$, a sample size of 30 patients would be powered to detect a clinically relevant difference of $\delta = 0.2$.

Accordingly, in this section we investigate the performance of the re-estimation procedures with the various design parameters motivated by those obtained from the results of this formoterol trial. In particular, this allows us to consider performance in a more challenging small sample setting. In all of what follows we set $\mu_0 = 1.51$, $\pi_2 = 0.03$, $\alpha = 0.1$ and $\beta = 0.2$, and we suppose that patients are allocated treatments via one of the six sequences listed above ($P = 2$). We then consider in turn the effect of varying one of the parameters σ_e^2, σ_b^2, and δ. As for Example 1, we will consider performance under the global null hypothesis ($\tau_1 = \tau_2 = 0$), when only treatment one is effective ($\tau_1 = \delta, \tau_2 = 0$), under the global alternative hypothesis ($\tau_1 = \tau_2 = \delta$), and under the observed treatment effects ($\tau_1 = 0.50, \tau_2 = 0.52$). Moreover, we again take $n_{\max} = 1000$, and estimate the average performance of each design and analysis procedure using 100,000 trial simulations.

S.M.5.2. Distributions of $\hat{\sigma}_e^2$, $\hat{\sigma}_b^2$ and $\hat{N}$

We first examine the performance of the re-estimation procedures when $\sigma_e^2 = 0.053$, $\sigma_b^2 = 0.49$, and $\delta = 0.2$, for $n_{\text{int}} = 18$. The resulting distributions of $\hat{\sigma}_e^2$, $\hat{\sigma}_b^2$, and $\hat{N}$, are shown in Supplementary Figures 9-11, via their median, lower and upper quartiles across the simulations. The results are grouped according to the true value of the treatment effects.

Following our findings for Example 1, the median values of $\hat{\sigma}_e^2$, $\hat{\sigma}_b^2$, and $\hat{N}$ for the unblinded and block randomised procedures are always close to their respective true values. However, this is not always the case for the adjusted estimators. In particular, the null adjusted estimator over-estimates the value of σ_e^2 when $\tau_1 = \delta$, and the alternative adjusted procedure under estimates σ_e^2 when $\tau_1 = \tau_2 = 0$. Both adjusted estimators perform extremely badly at re-estimating σ_e^2 under the observed treatment effects.

In terms of $\hat{\sigma}_b^2$, the performance of the re-estimation procedures is more comparable, though the adjusted procedures fair worse under the observed treatment effects.

As expected, the results for $\hat{N}$ once again mirror those for $\hat{\sigma}_e^2$. This means, in particular, that the

median value of $\hat{N}$ is substantially larger for the adjusted estimators under the observed treatment effects, and is slightly larger for the null adjusted procedure when $\tau_1 = \delta$.

Increasing the value of n_{int} reduces the interquartile range for $\hat{\sigma}_e^2$ and $\hat{N}$ for each procedure, and results in median values closer to the truth, as would be expected. Finally, we observe that the interquartile range for the unblinded procedure is often smaller than that of its adjusted or block randomisation counterparts.

S.M.5.3. Familywise error-rate, power, and sample size inflation factor

For the scenarios from Section S.M.5.2 that were not conducted under the observed treatment effects, the estimated FWER and power were also recorded. Additional simulations were also performed to ascertain the power of the procedures under the global alternative hypothesis when the sample size inflation factor introduced in Section 3.7 was utilised. The results are displayed in Supplementary Table 1.

We can see that the unblinded and block randomised procedures experience similar and substantial inflation to the FWER. In contrast the adjusted procedures have an equal FWER with much smaller inflation above the nominal level. Thus for this example, the small requisite sample size appears to have inhibited the ability of the re-estimation procedures to retain a FWER close to α.

Each of the procedures attain the desired power apart from that utilising the alternative adjusted estimator. The null adjusted estimator provides the largest power. These findings are not surprising in light of the distributions of $\hat{\sigma}_e^2$ observed in Supplementary Figure 9. Therefore in this instance the inflation factor appears to only be of use to the alternative adjusted procedure.

Supplementary Table 1: The estimated familywise error-rate (FWER) is shown for each of the considered re-estimation procedures under the global null hypothesis, for Example 2. Corresponding values of the power when only treatment one is effective, under the global alternative hypothesis when both experimental treatments are effective, and under the global alternative hypothesis with use of the sample size inflation factor, are also shown. The Monte Carlo error of the FWER and power values is approximately 0.001 and 0.0013 respectively in each instance. All figures are given to four decimal places.

		Power		
Re-estimation procedure	FWER	$\boldsymbol{\tau} = (\delta, 0)$	$\boldsymbol{\tau} = (\delta, \delta)$	$\boldsymbol{\tau} = (\delta, \delta)$ with Inf. Fac.
Unblinded	0.1174	0.8027	0.8047	0.8276
Null Adjusted	0.1069	0.8204	0.8186	0.8450
Alt. Adjusted	0.1069	0.7490	0.7485	0.7710
Block rand. with $n_B = 3$	0.1157	0.8013	0.8029	0.8273

S.M.5.4. Influence of σ_e^2

We now consider cases where $\sigma_b^2 = 0.49$ and $\delta = 0.2$, but $\sigma_e^2 \neq 0.053$. Specifically, in Supplementary Figures 12 and 13 we respectively examine the FWER and power of the re-estimation procedures under the global null and alternative hypotheses when $\sigma_e^2 \in [0.25(0.053), 4(0.053)]$.

Supplementary Figure 13 implies that the results for Example 2 are similar to those observed for Example 1. Explicitly, when σ_e^2 is very small the procedures are each over-powered, and as σ_e^2 increases there appears to be less of an effect upon the power.

In contrast, the results on the FWER displayed in Supplementary Figure 12 depict a distinctive pattern for each of the re-estimation procedures. Most likely, the observed peaks reflect a point at which the designs begin to switch from terminating the trial at the interim reassessment, to continuing to the end of the second stage. Overall, it is clear that the adjusted procedures typically have similar values of the FWER, with the same true of the unblinded and block randomised procedures. Moreover, whilst

all of the estimators experience notable inflation to the FWER, it is smaller for the adjusted estimators, particularly as σ_e^2 increases.

S.M.5.5. Influence of σ_b^2

Next, we examine scenarios where $\sigma_e^2 = 0.053$ and $\delta = 0.2$, but $\sigma_b^2 \neq 0.49$: in Supplementary Figures 14 and 15 we respectively examine the FWER and power of the re-estimation procedures under the global null and alternative hypotheses when $\sigma_b^2 \in [0.25(0.49), 4(0.49)]$.

Allowing for Monte Carlo error, it appears that the value of σ_b^2, as in Example 1, has negligible effect upon the FWER and power of each of the the re-estimation procedures. This may seem surprising as we specifically noted that a value for σ_b^2 would be required to estimate the required sample size of a trial using the incomplete block sequences specified in Section S.M.5.1. However, this result reflects two factors. The first is that, as evidenced by Supplementary Figure 10, the re-estimation procedures are very effective at estimating the value of σ_b^2. More importantly, though, is that whilst the sample size required by the formoterol trial will be dependent upon σ_b^2, it will still be principally driven by the value of σ_e^2. Therefore, the main driver of the utility of the re-estimation procedures in this setting remains their ability to re-estimate the within person variance.

S.M.5.6. Influence of δ

In our final investigations for Example 2 we consider scenarios in which $\sigma_e^2 = 0.053$ and $\sigma_b^2 = 0.49$, but $\delta \neq 0.2$. Precisely, in Supplementary Figures 16 and 17 we respectively examine the FWER and power of the re-estimation procedures under the global null and alternative hypotheses when $\delta \in [0.5(0.2), 2(0.2)]$.

Examining Supplementary Figure 16, the FWER for the unblinded and block randomised procedures display the same rising and falling shape as observed in Supplementary Figure 12. In contrast, the FWER for the adjusted procedures appears only to rise with in δ. This should not be surprising as larger values of δ imply smaller requisite sample sizes, leaving the procedures prone to small sample size issues.

From Supplementary Figure 17, as we would anticipate, for the larger considered values of δ the re-estimation procedures are substantially over-powered. All but the alternative adjusted procedure still perform well though when $\delta = 0.1$.

S.M.6. Example 3: Hypertension

S.M.6.1. Introduction

Ebbutt (1984) presented an analysis of data from a two-treatment ($D = 2$) three-period ($P = 3$) crossover hypertension trial. Denoting the treatments by $d = 0, 1$, patients were assigned to one of the following four sequence groups

$$011,\ 100,\ 010,\ 101.$$

Whilst Ebbutt (1984) assessed the data from only ten subjects on each sequence, Jones and Kenward (2014) discussed the data from all patients who completed the trial. Here, we suppose that the data Jones and Kenward (2014) presented on the outcome systolic blood pressure were to be analysed using the linear mixed model (1) in order to test the following hypotheses

$$H_{01} : \tau_1 \geq 0, \qquad H_{11} : \tau_1 < 0.$$

Thus, as in Example 1, a negative treatment effect implies efficacy.

In analysing the data from this trial using linear mixed model (1) we find

$$\hat{\mu}_0 = 156.77,\ \hat{\pi}_2 = -2.13,\ \hat{\pi}_3 = -4.90,\ \hat{\tau}_1 = -7.55,\ \hat{\sigma}_e^2 = 169.8,\ \hat{\sigma}_b^2 = 255.0.$$

Therefore, for $\alpha = 0.025$, the trial's sample size of 90 patients would provide power for a clinically relevant difference of $\delta = -5.39$ when $\beta = 0.1$.

In this section, we explore the performance of the re-estimation procedures with the parameters motivated by the results of this hypertension trial. Thus, having considered complete block sequences in Example 1, and incomplete block sequences in Example 2, we now consider a design utilising extra-period sequences.

Following the same path as in Example 2, throughout what follows we set $\mu_0 = 156.77$, $\pi_2 = -2.13$, $\pi_3 = -4.90$, $\alpha = 0.025$ and $\beta = 0.1$, supposing that patients are allocated treatments based upon one of the four sequences given above. We then again consider the effect of varying the parameters σ_e^2, σ_b^2, and δ. We examine performance under the null hypothesis ($\tau_1 = 0$), the alternative hypothesis ($\tau_1 = \delta$), and under the observed treatment effect ($\tau_1 = -7.55$). Finally, once more, we take $n_{\text{max}} = 1000$, and estimate the average performance of each design and analysis procedure using 100,000 trial simulations.

S.M.6.2. Distributions of $\hat{\sigma}_e^2$, $\hat{\sigma}_b^2$ and $\hat{N}$

We first examine the performance of the re-estimation procedures when $\sigma_e^2 = 169.8$, $\sigma_b^2 = 255.0$, and $\delta = -5.39$, for $n_{\text{int}} \in \{16, 32, 48\}$. The resulting distributions of $\hat{\sigma}_e^2$, $\hat{\sigma}_b^2$, and $\hat{N}$, are shown in Supplementary Figures 18-20, via their median, lower and upper quartiles across the simulations. The results are grouped according to the timing of the re-estimation and by the true value of the treatment effects.

Overall, the results are very similar to those of Examples 1 and 2. In particular, the unblinded and block randomised procedures typically perform well in terms of re-estimating σ_e^2, whilst the adjusted estimators performance is highly dependent on the value of τ_1.

As in Example 1, increasing the value of n_B for the block randomised procedure appears advantageous. Moreover, as expected, increasing the value of n_{int} reduces the distance between the median values of $\hat{\sigma}_e^2$, $\hat{\sigma}_b^2$, and $\hat{N}$ and their respective true values, whilst also reducing the interquartile range.

In this case, the distributions of the $\hat{\sigma}_b^2$ are comparable across the procedures, regardless of the value of n_{int} or τ_1.

Once more, the results for $\hat{N}$ largely reflect those for $\hat{\sigma}_e^2$. However, the median values for $\hat{N}$ are larger when using the block randomised procedure with $n_B = 8$. This is a consequence of the fact that by our assumptions an entire additional block of patients will be recruited even when a smaller number of patients are required to attain the requisite sample size.

S.M.6.3. Familywise error-rate and power

For the scenarios from Section S.M.6.2 that were not conducted under the observed treatment effect, the estimated FWER and power were also recorded. The results are displayed in Supplementary Table 2.

In this instance, we observe that the FWER is in many instances slightly below the nominal level, with a maximal value of only 0.0252 for the block randomised procedure with $n_B = 4$ when $n_{\text{int}} \in \{16, 48\}$. This is likely a result of the fact that the number of observations accrued in each of the designs is, for a crossover trial at least, large relative to the number of hypotheses that are to be tested.

Most of the re-estimation procedures attain a power close to the nominal level. However, the alternative adjusted estimator again leads to lower levels of power. Finally, as would be anticipated, increasing the value of n_{int} improves the power of the re-estimation procedures. However, it has no clear effect upon the FWER.

Supplementary Table 2: The estimated familywise error-rate (FWER) and power is shown for each of the considered re-estimation procedures and several values of n_{int} under the null and alternative hypotheses respectively, for Example 3. The Monte Carlo error of the FWER and power values is approximately 0.0005 and 0.001 respectively in each instance. All figures are given to four decimal places.

Re-estimation procedure	n_{int}	FWER	Power
Unblinded	16	0.0243	0.8761
Null Adjusted	16	0.0243	0.8758
Alt. Adjusted	16	0.0237	0.8517
Block rand. with $n_B = 4$	16	0.0252	0.8696
Unblinded	32	0.0247	0.8913
Null Adjusted	32	0.0239	0.8895
Alt. Adjusted	32	0.0240	0.8712
Block rand. with $n_B = 4$	32	0.0247	0.8876
Block rand. with $n_B = 8$	32	0.0241	0.8942
Unblinded	48	0.0246	0.8961
Null Adjusted	48	0.0242	0.8946
Alt. Adjusted	48	0.0242	0.8744
Block rand. with $n_B = 4$	48	0.0252	0.8946
Block rand. with $n_B = 8$	48	0.0249	0.9007

S.M.6.4. Influence of σ_e^2

We now consider cases where $\sigma_b^2 = 255.0$, and $\delta = -5.39$, but $\sigma_e^2 \neq 169.8$. Specifically, in Supplementary Figures 21 and 22 we respectively examine the FWER and power of the re-estimation procedures under the null and alternative hypotheses when $\sigma_e^2 \in [0.25(169.8), 4(169.8)]$.

Here, there appears to be some evidence that the smallest considered values of σ_e^2 result in lower values of the FWER. This may once more reflect a change between terminating the trial at the interim reassessment and continuing to the end of the second stage. Nonetheless, is it clear that in this case the procedures control the FWER well regardless of the value of the within person variance.

Similarly, whilst the smallest values of σ_e^2 lead to the designs being substantially over-powered, it is evident that when σ_e^2 is increased they are still able to provide approximately the desired power.

S.M.6.5. Influence of σ_b^2

Next, we examine scenarios in which $\sigma_e^2 = 169.8$, and $\delta = -5.39$, but $\sigma_b^2 \neq 255.0$: in Supplementary Figures 23 and 24 we respectively examine the FWER and power of the re-estimation procedures under the null and alternative hypotheses when $\sigma_b^2 \in [0.25(255.0), 4(255.0)]$.

Similar to both Examples 1 and 2, allowing for Monte Carlo error, the value of σ_b^2 seems to have little effect upon the ability of the re-estimation procedures to control the FWER to 0.025, and to provide power at level 0.9. We therefore have further evidence that we do not need to be concerned about the underlying between patient variance when considering the appropriate use of the proposed methods.

S.M.6.6. Influence of δ

We now investigate settings in which $\sigma_e^2 = 169.8$ and $\sigma_b^2 = 255.0$, but $\delta \neq -5.39$. Precisely, in Supplementary Figures 25 and 26 we respectively examine the FWER and power of the re-estimation procedures under the null and alternative hypotheses when $\delta \in [2(-5.39), 0.5(-5.39)]$.

Our results indicate that in this case, the smallest considered values of δ appear to result in reduced values of the FWER. However, regardless of the value of δ, each of the procedures is generally able to control the FWER to approximately the nominal level.

From Supplementary Figure 26, we once more observe that when the magnitude of δ is large, the designs are over-powered, but outside of this region the specified clinically relevant difference appears to have little effect upon the power.

S.M.6.7. Sample size inflation factor

Our final consideration is to once more examine the utility of the sample size inflation factor introduced in Section 3.7. In Supplementary Figure 27 we present the power of the re-estimation procedures under the alternative hypothesis, when $n_{\text{int}} \in \{16, 32, 48\}$, with and without the use of the inflation factor.

As was observed for Example 1, the inflation factor leads to a notable boost in power, helping several of the estimators achieve the desired performance. Therefore, when there are concerns around attaining the desired power, we may again recommend that investigators consider utilising this simple adjustment.

References

Ebbutt, A. F. (1984) Three-period crossover designs for two treatments. *Biometrics* **40:**347-351.

Jones, B., and Kenward, M. G. (2014) *Design and Analysis of Cross-Over Trials.* Chapman & Hall, Boca Raton.

Senn, S. J. (2002) *Cross-over Trials in Clinical Research.* John Wiley & Sons, Chichester.

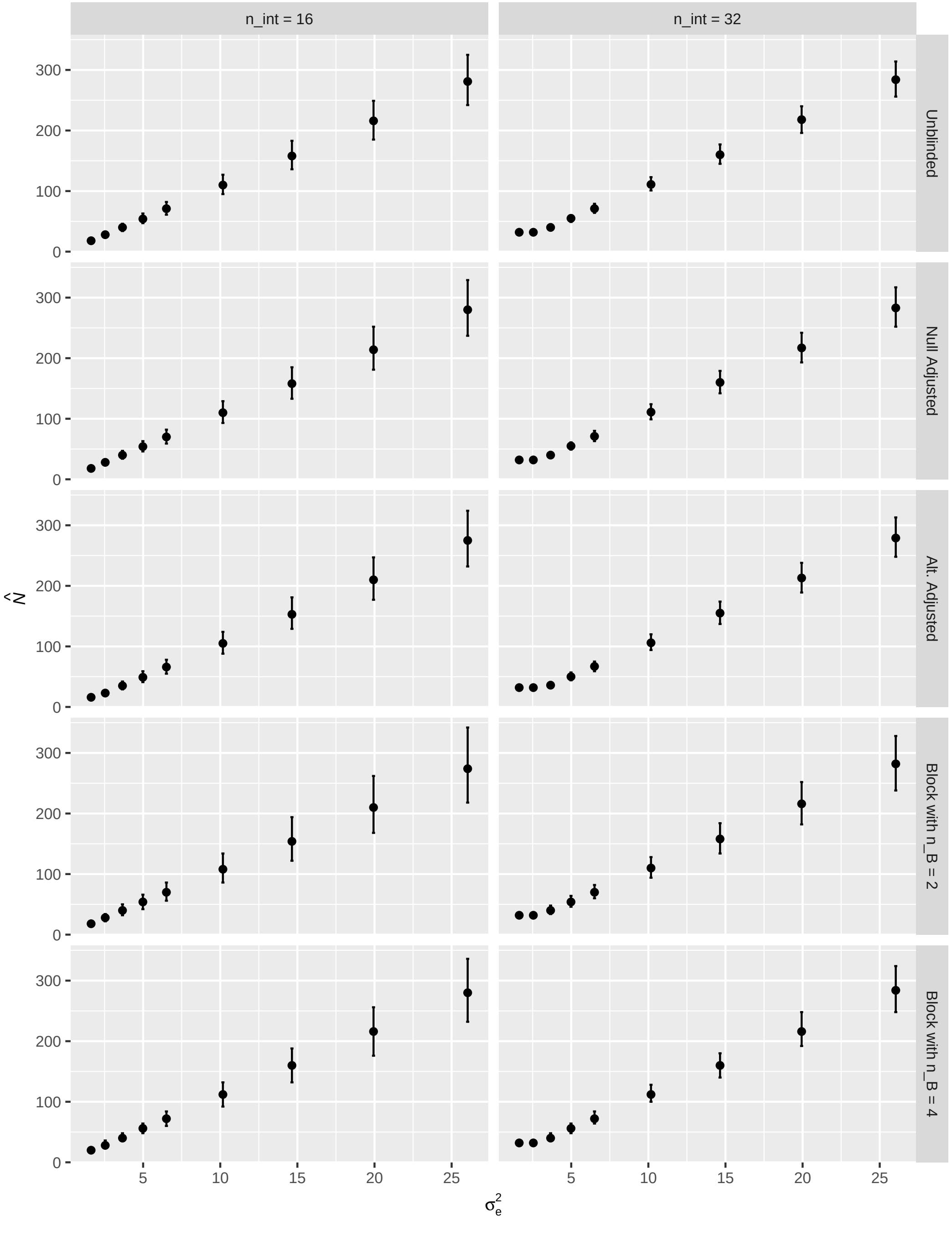

Supplementary Figure 1: The lower, median, and upper quartile values of the re-estimated required sample size, $\hat{N}$, is shown under the global null hypothesis for each of the re-estimation procedures when $n_{\text{int}} \in \{16, 32\}$, as a function of the within person variance σ_e^2, for Example 1.

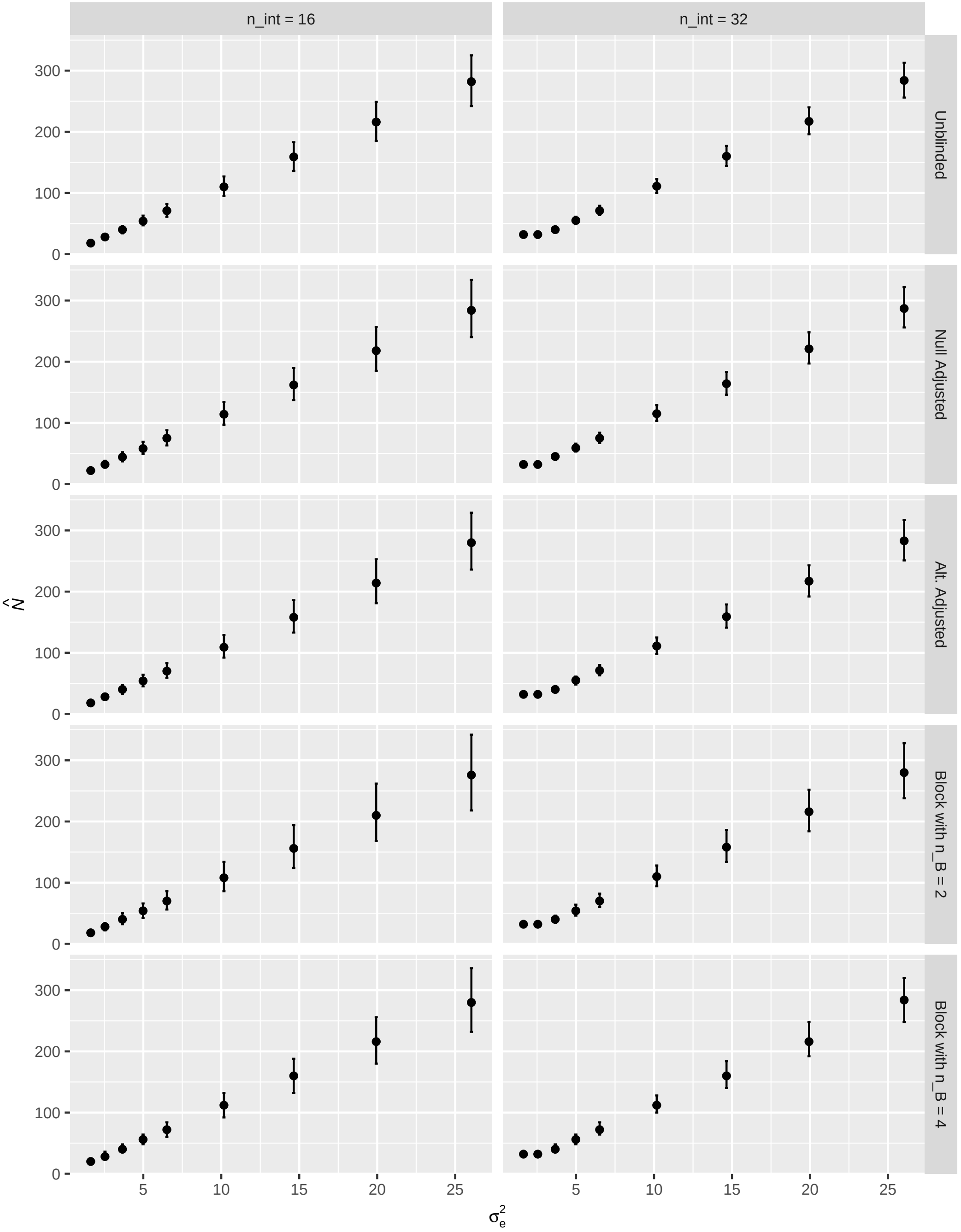

Supplementary Figure 2: The lower, median, and upper quartile values of the re-estimated required sample size, $\tilde{N}$, is shown under the global alternative hypothesis for each of the re-estimation procedures when $n_{\text{int}} \in \{16, 32\}$, as a function of the within person variance σ_e^2, for Example 1.

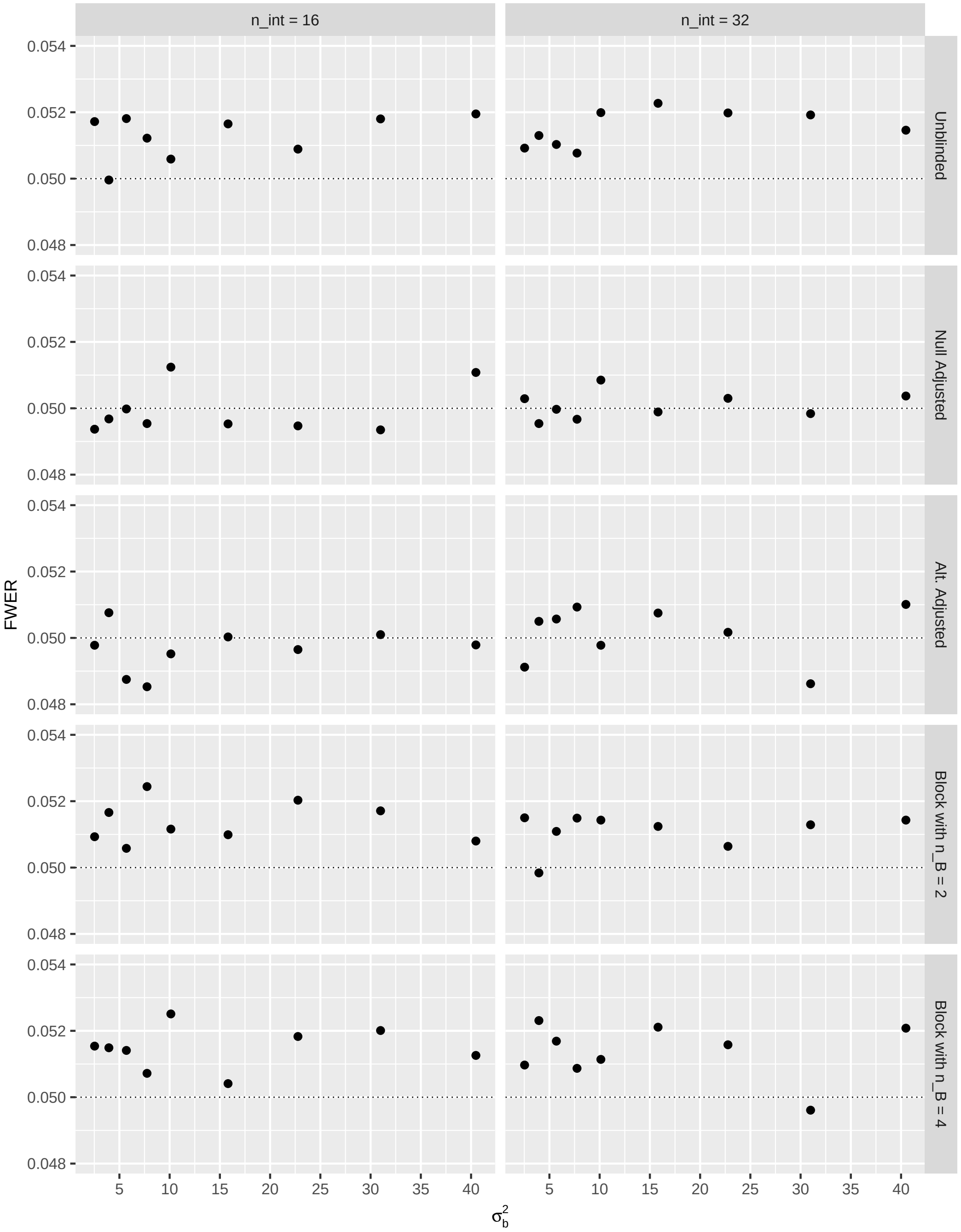

Supplementary Figure 3: The simulated familywise error-rate (FWER) is shown under the global null hypothesis for each of the re-estimation procedures when $n_{\text{int}} \in \{16, 32\}$, as a function of the between person variance σ_b^2, for Example 1. The Monte Carlo error is approximately 0.0007 in each instance. The dashed line indicates the desired value of the FWER.

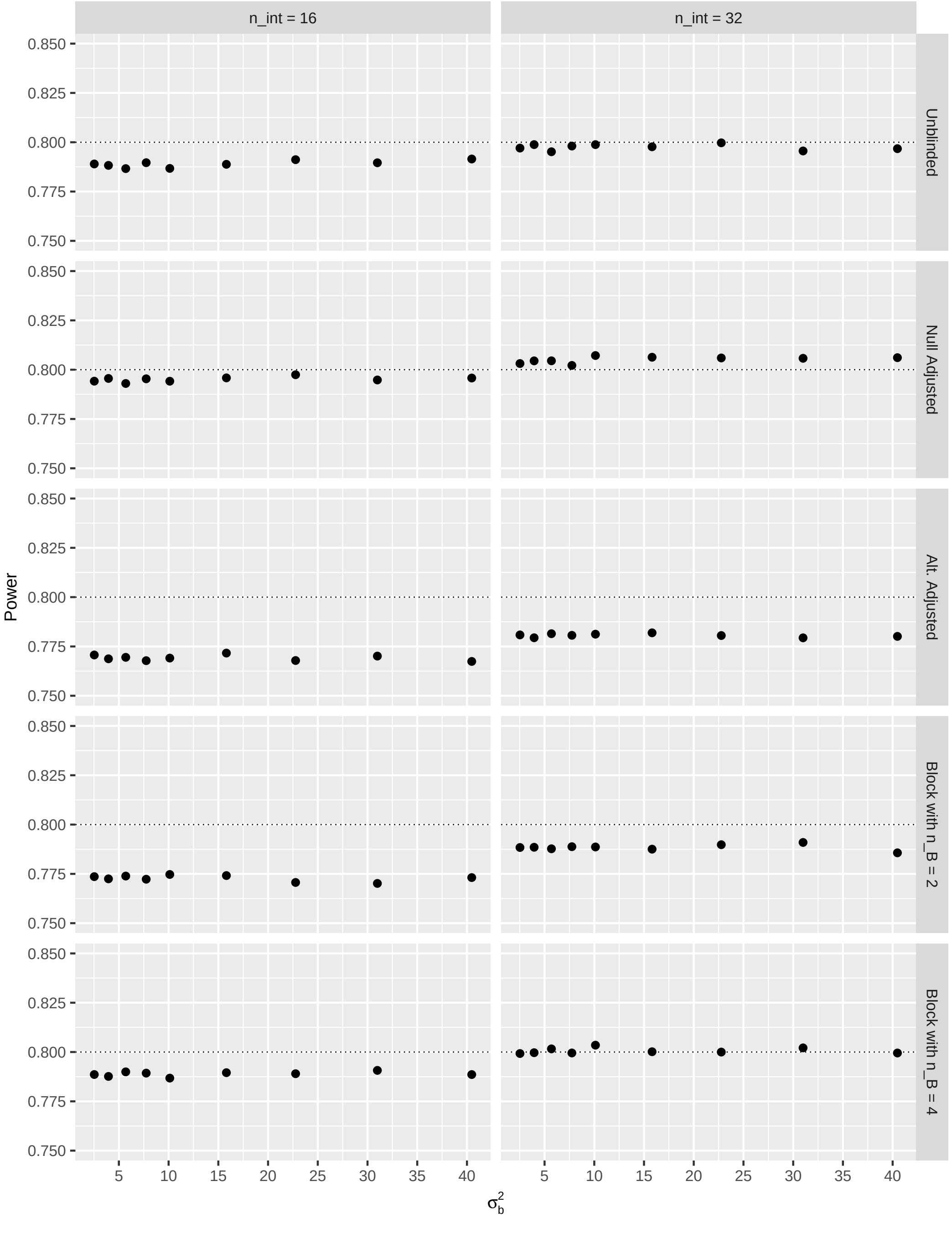

Supplementary Figure 4: The simulated power is shown under the global alternative hypothesis for each of the re-estimation procedures when $n_{\text{int}} \in \{16, 32\}$, as a function of the within person variance σ_b^2, for Example 1. The Monte Carlo error is approximately 0.0013 in each instance. The dashed line indicates the desired value of the power.

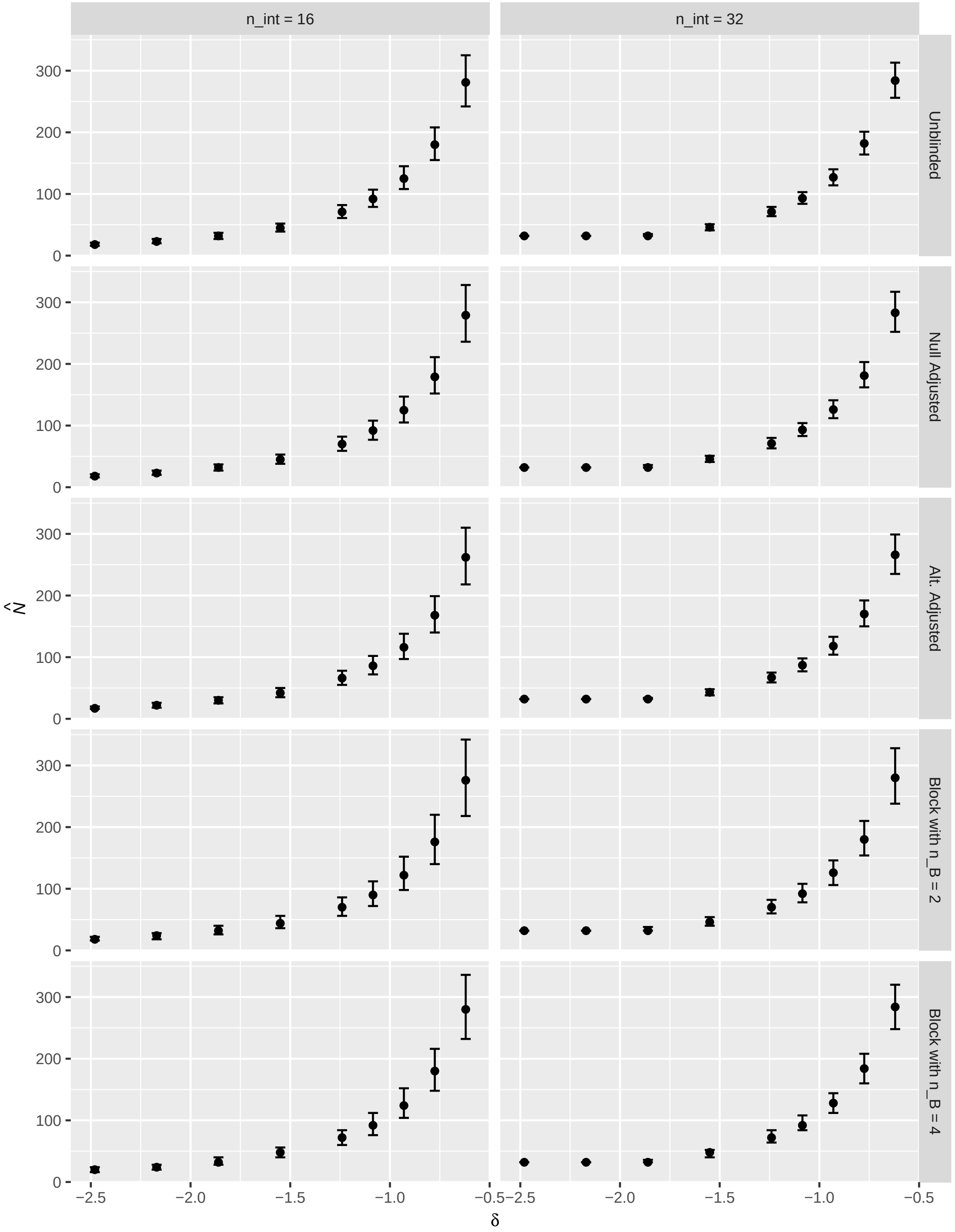

Supplementary Figure 5: The lower, median, and upper quartile values of the re-estimated required sample size, $\hat{N}$, is shown under the global null hypothesis for each of the re-estimation procedures when $n_{\text{int}} \in \{16, 32\}$, as a function of the clinically relevant difference δ, for Example 1.

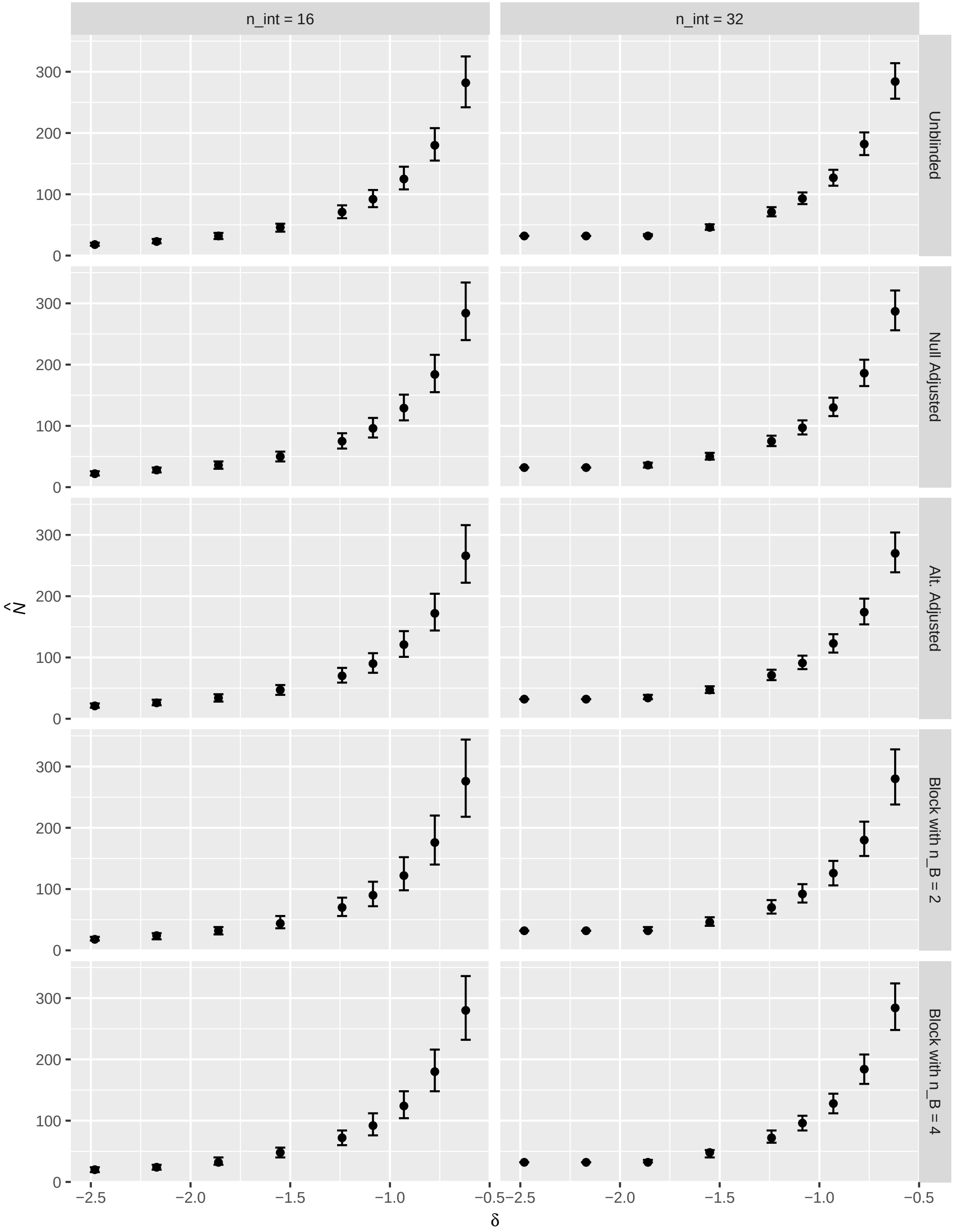

Supplementary Figure 6: The lower, median, and upper quartile values of the re-estimated required sample size, $\tilde{N}$, is shown under the global alternative hypothesis for each of the re-estimation procedures when $n_{\text{int}} \in \{16, 32\}$, as a function of the clinically relevant difference δ, for Example 1.

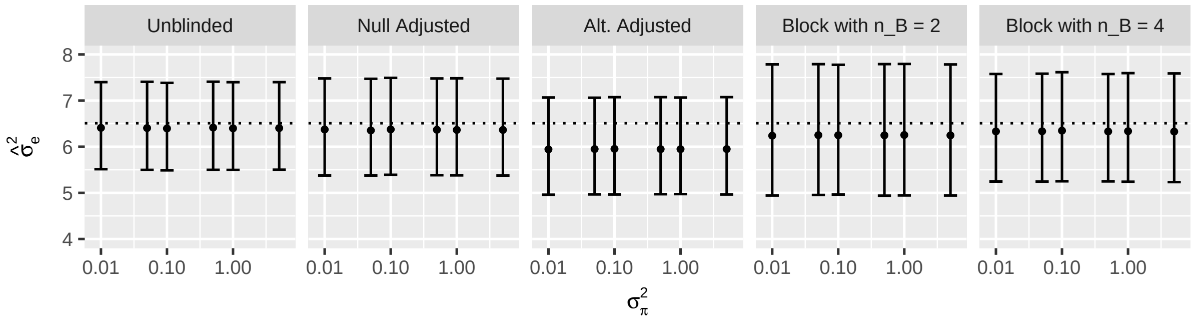

Supplementary Figure 7: The distribution of $\hat{\sigma}_e^2$ is shown under the global null hypothesis for each of the re-estimation procedures when $n_{\text{int}} = 16$, as a function of the variance of the period effects σ_π^2, for Example 1. Precisely, for each scenario, the median, lower and upper quartile values of $\hat{\sigma}_e^2$ across the simulations are given. The dashed line indicates the true value of σ_e^2.

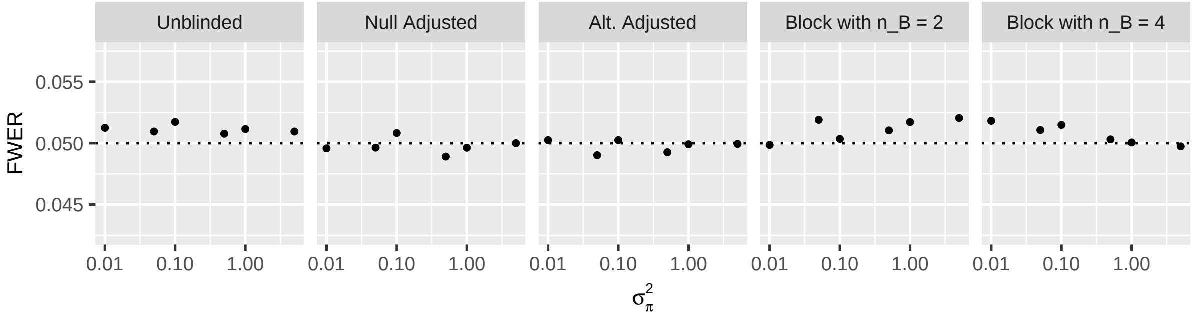

Supplementary Figure 8: The simulated familywise error-rate (FWER) is shown under the global null hypothesis for each of the re-estimation procedures when $n_{\text{int}} = 16$, as a function of the variance of the period effects σ_π^2, for Example 1. The Monte Carlo error is approximately 0.0007 in each instance. The dashed line indicates the desired value of the FWER.

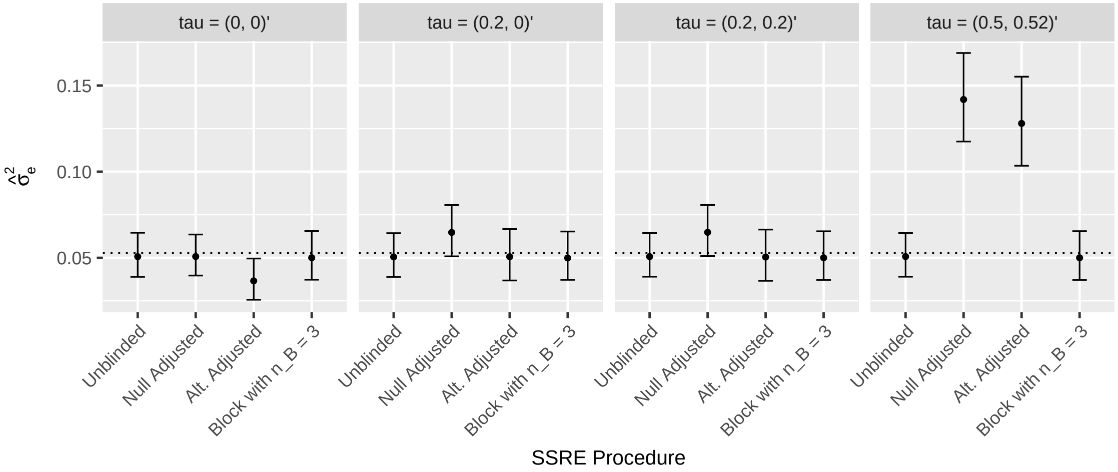

Supplementary Figure 9: The distribution of $\hat{\sigma}_e^2$ is shown for each of the re-estimation procedures for several values of $\boldsymbol{\tau}$, for Example 2. Precisely, for each scenario, the median, lower and upper quartile values of $\hat{\sigma}_e^2$ across the simulations are given. The dashed line indicates the true value of σ_e^2.

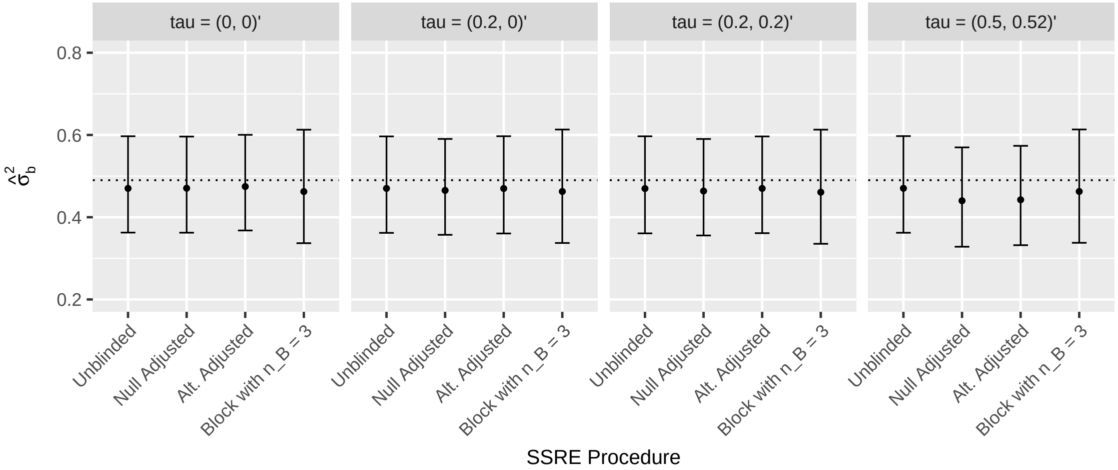

Supplementary Figure 10: The distribution of $\hat{\sigma}_b^2$ is shown for each of the re-estimation procedures for several values of $\boldsymbol{\tau}$, for Example 2. Precisely, for each scenario, the median, lower and upper quartile values of $\hat{\sigma}_b^2$ across the simulations are given. The dashed line indicates the true value of σ_b^2.

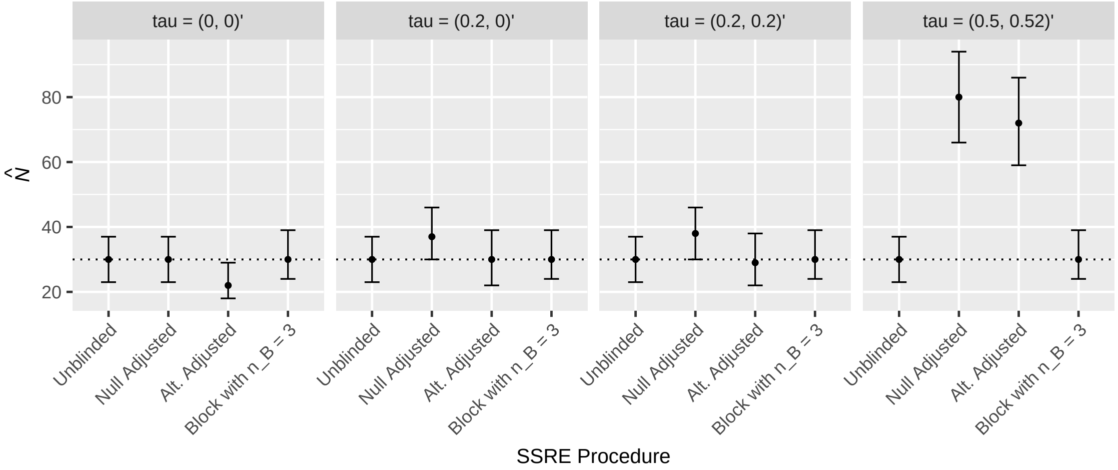

Supplementary Figure 11: The distribution of $\hat{N}$ is shown for each of the re-estimation procedures for several values of $\boldsymbol{\tau}$, for Example 2. Precisely, for each scenario, the median, lower and upper quartile values of $\hat{N}$ across the simulations are given. The dashed line indicates the true required value of N.

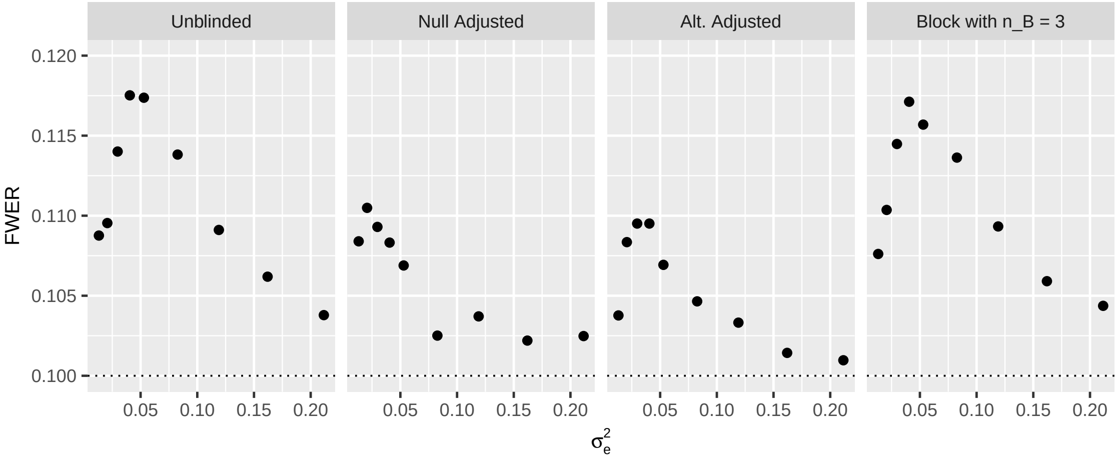

Supplementary Figure 12: The simulated familywise error-rate (FWER) is shown under the global null hypothesis for each of the re-estimation procedures, as a function of the within person variance σ_e^2, for Example 2. The Monte Carlo error is approximately 0.001 in each instance. The dashed line indicates the desired value of the FWER.

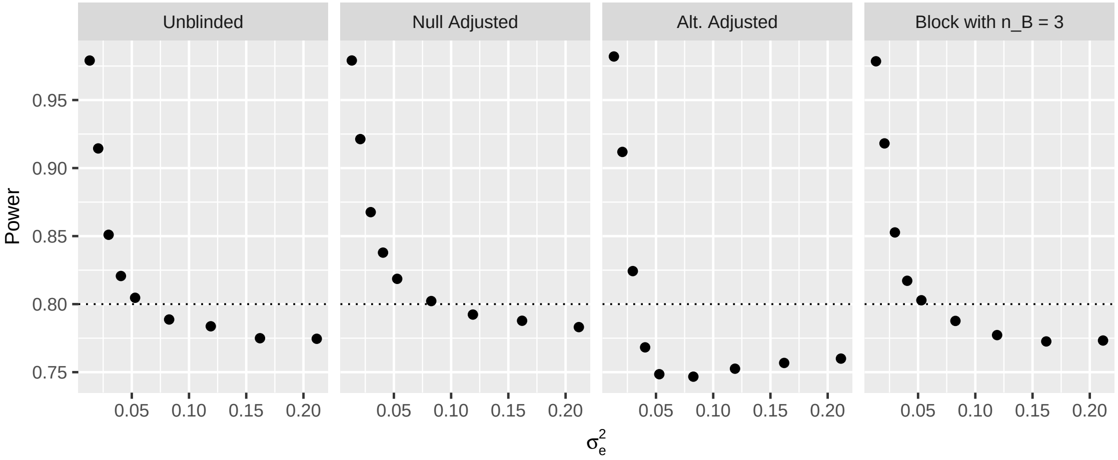

Supplementary Figure 13: The simulated power is shown under the global alternative hypothesis for each of the re-estimation procedures, as a function of the within person variance σ_e^2, for Example 2. The Monte Carlo error is approximately 0.0013 in each instance. The dashed line indicates the desired value of the power.

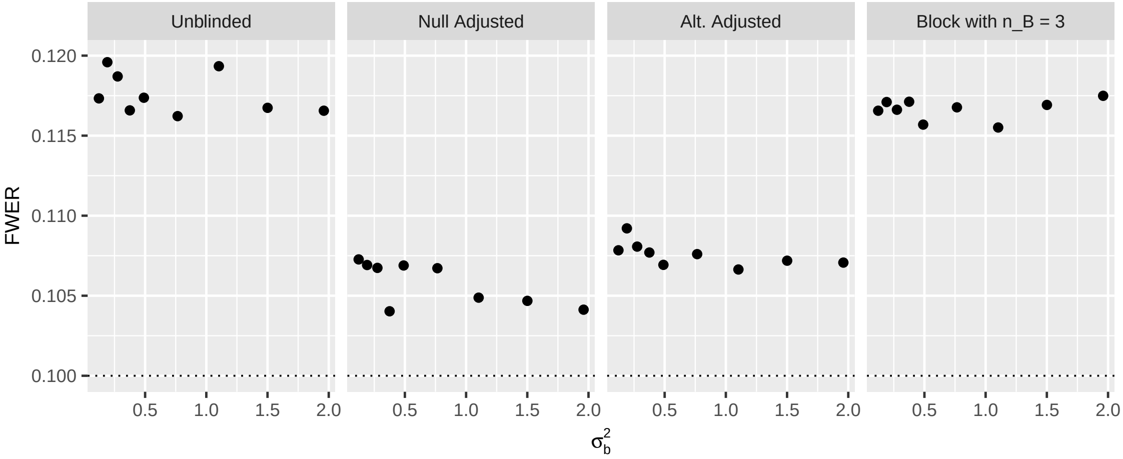

Supplementary Figure 14: The simulated familywise error-rate (FWER) is shown under the global null hypothesis for each of the re-estimation procedures, as a function of the within person variance σ_b^2, for Example 2. The Monte Carlo error is approximately 0.001 in each instance. The dashed line indicates the desired value of the FWER.

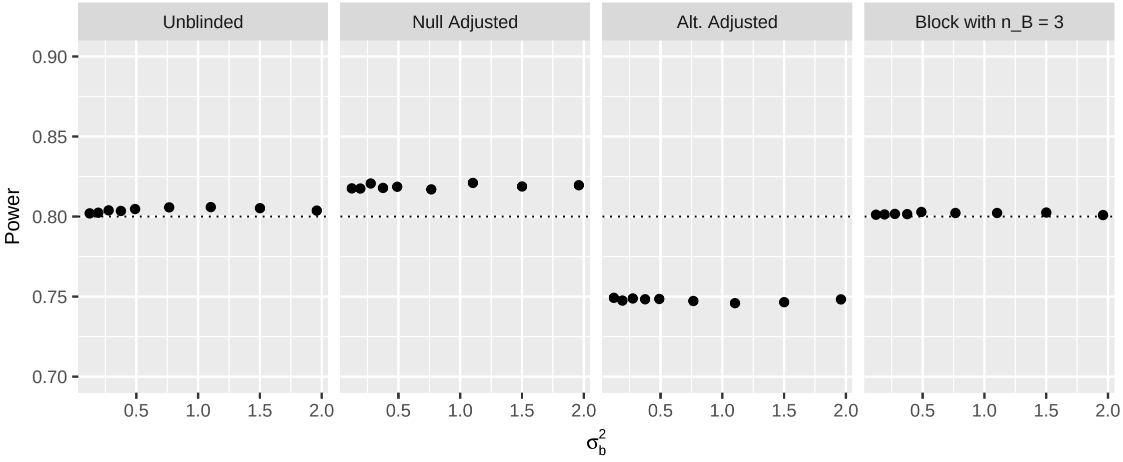

Supplementary Figure 15: The simulated power is shown under the global alternative hypothesis for each of the re-estimation procedures, as a function of the within person variance σ_b^2, for Example 2. The Monte Carlo error is approximately 0.0013 in each instance. The dashed line indicates the desired value of the power.

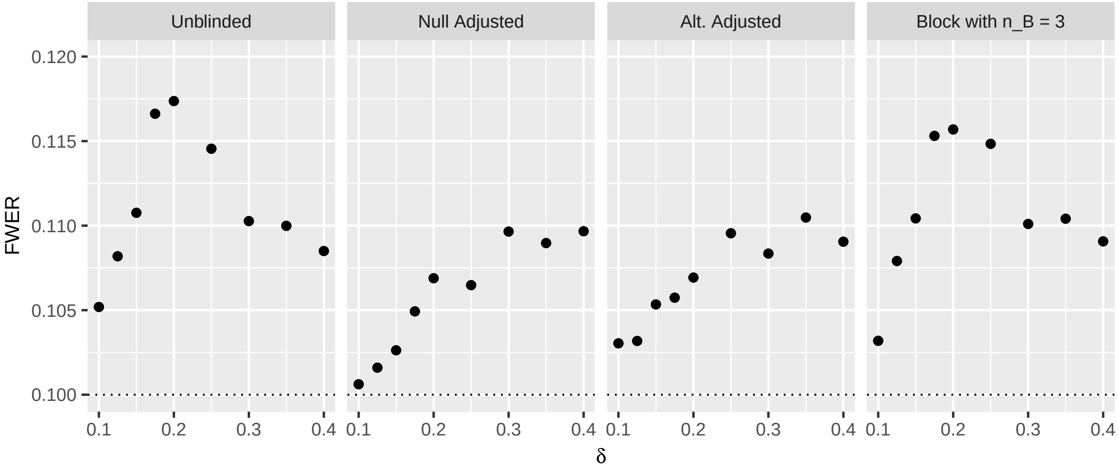

Supplementary Figure 16: The simulated familywise error-rate (FWER) is shown under the global null hypothesis for each of the re-estimation procedures, as a function of the clinically relevant difference δ, for Example 2. The Monte Carlo error is approximately 0.001 in each instance. The dashed line indicates the desired value of the FWER.

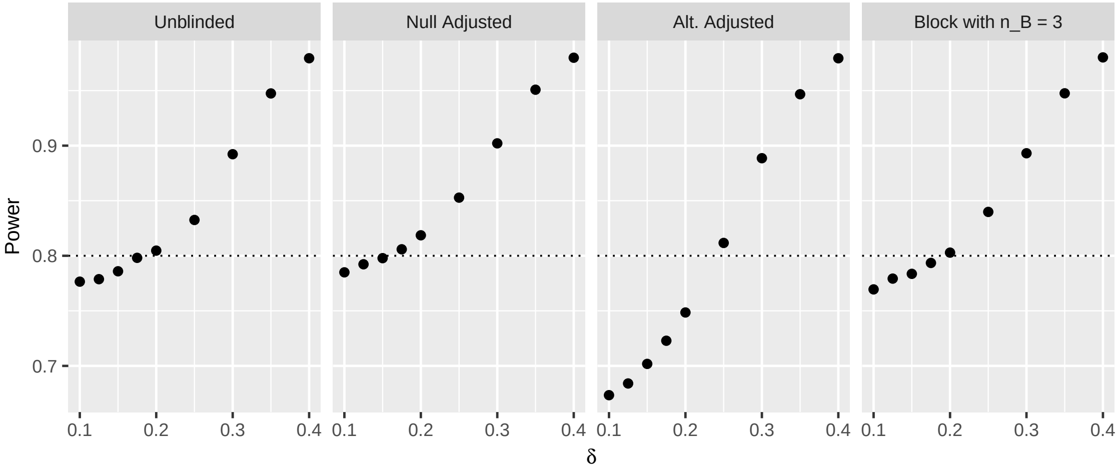

Supplementary Figure 17: The simulated power is shown under the global alternative hypothesis for each of the re-estimation procedures, as a function of the clinically relevant difference δ, for Example 2. The Monte Carlo error is approximately 0.0013 in each instance. The dashed line indicates the desired value of the power.

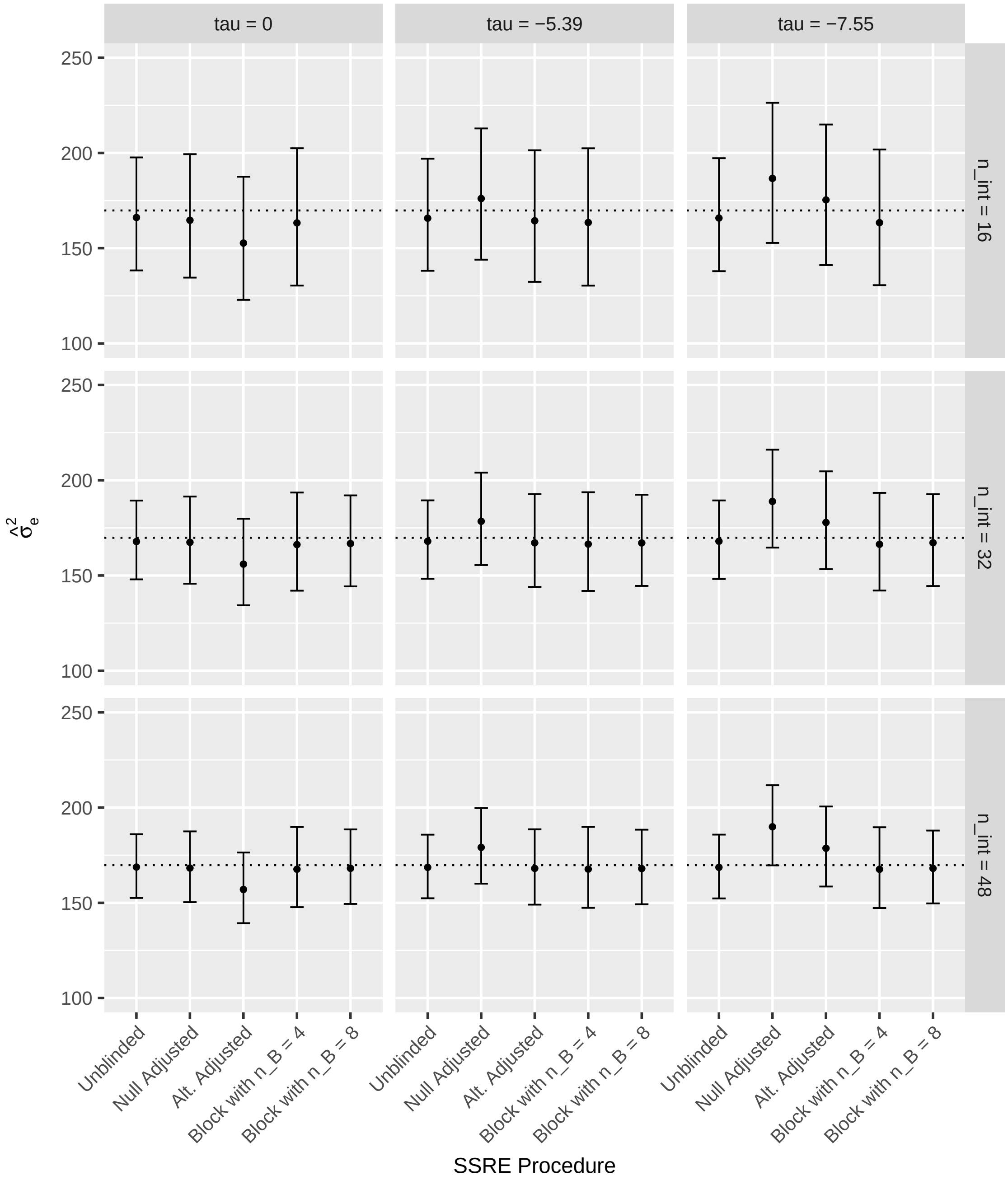

Supplementary Figure 18: The distribution of $\hat{\sigma}_e^2$ is shown for each of the re-estimation procedures for several values of $\boldsymbol{\tau}$, and for several values of n_{int}, for Example 3. Precisely, for each scenario, the median, lower and upper quartile values of $\hat{\sigma}_e^2$ across the simulations are given. The dashed line indicates the true value of σ_e^2.

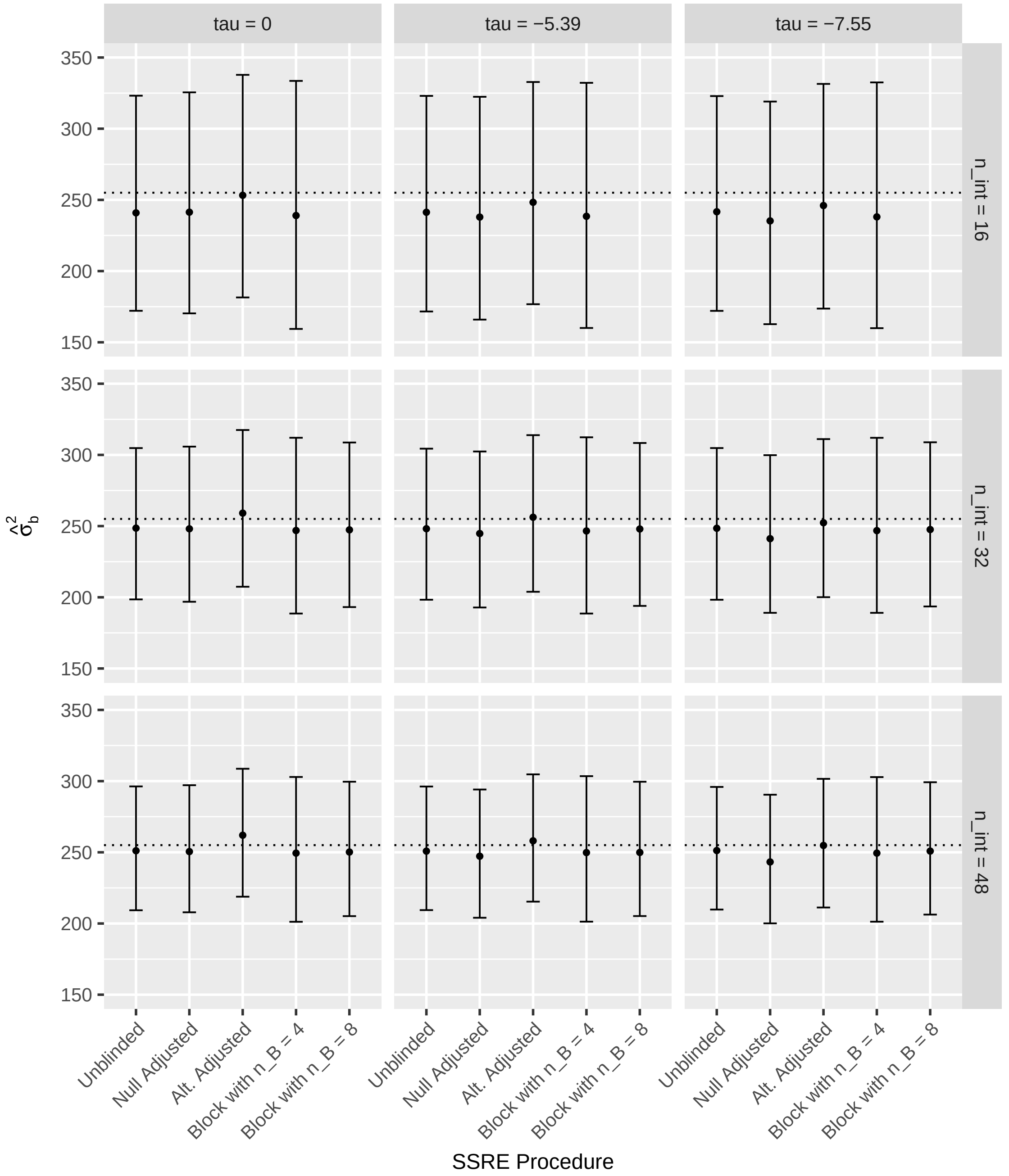

Supplementary Figure 19: The distribution of $\hat{\sigma}_b^2$ is shown for each of the re-estimation procedures for several values of $\boldsymbol{\tau}$, and several values of n_{int}, for Example 3. Precisely, for each scenario, the median, lower and upper quartile values of $\hat{\sigma}_b^2$ across the simulations are given. The dashed line indicates the true value of σ_b^2.

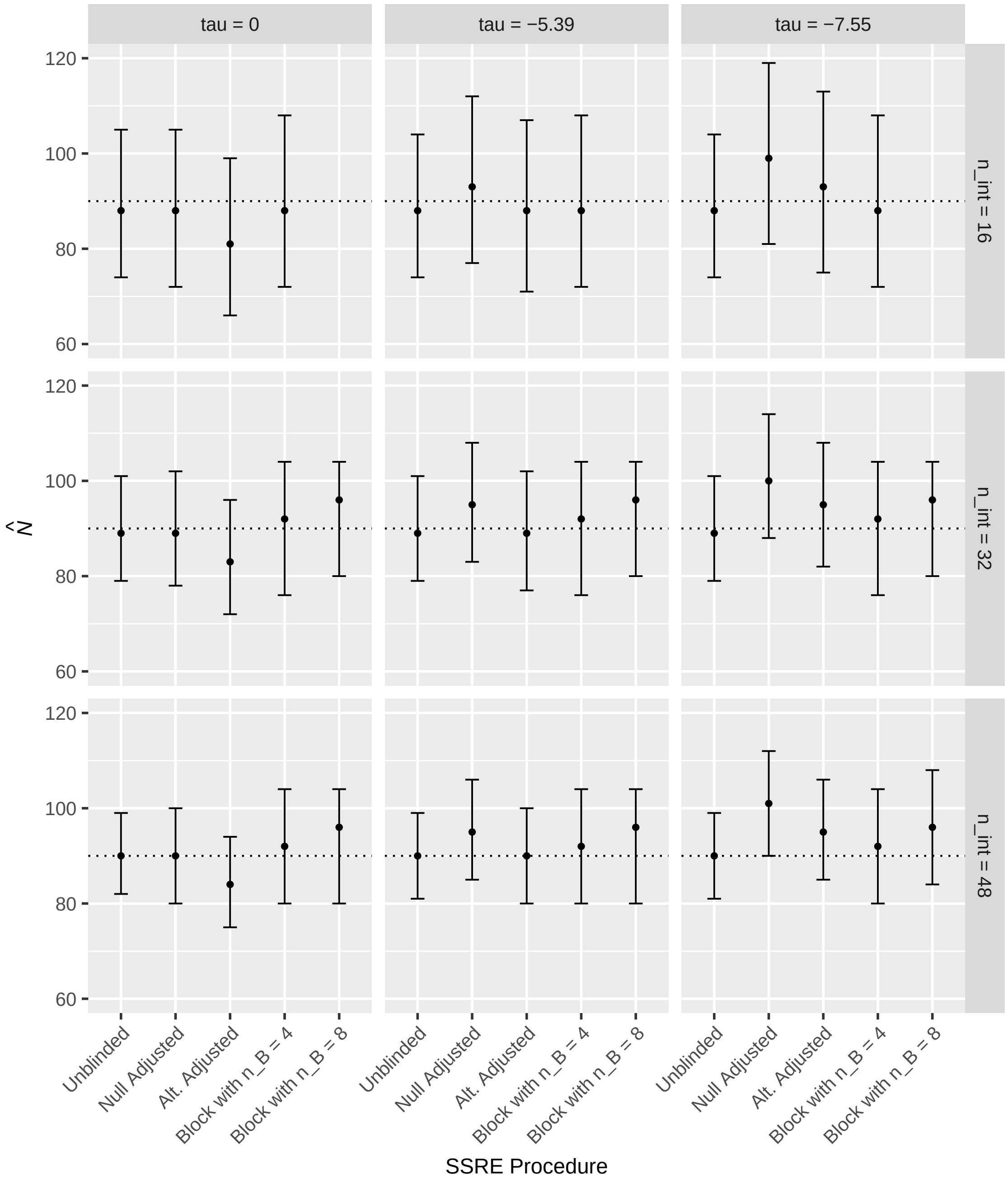

Supplementary Figure 20: The distribution of $\hat{N}$ is shown for each of the re-estimation procedures for several values of $\boldsymbol{\tau}$, and several values of n_{int}, for Example 3. Precisely, for each scenario, the median, lower and upper quartile values of $\hat{N}$ across the simulations are given. The dashed line indicates the true required value of N.

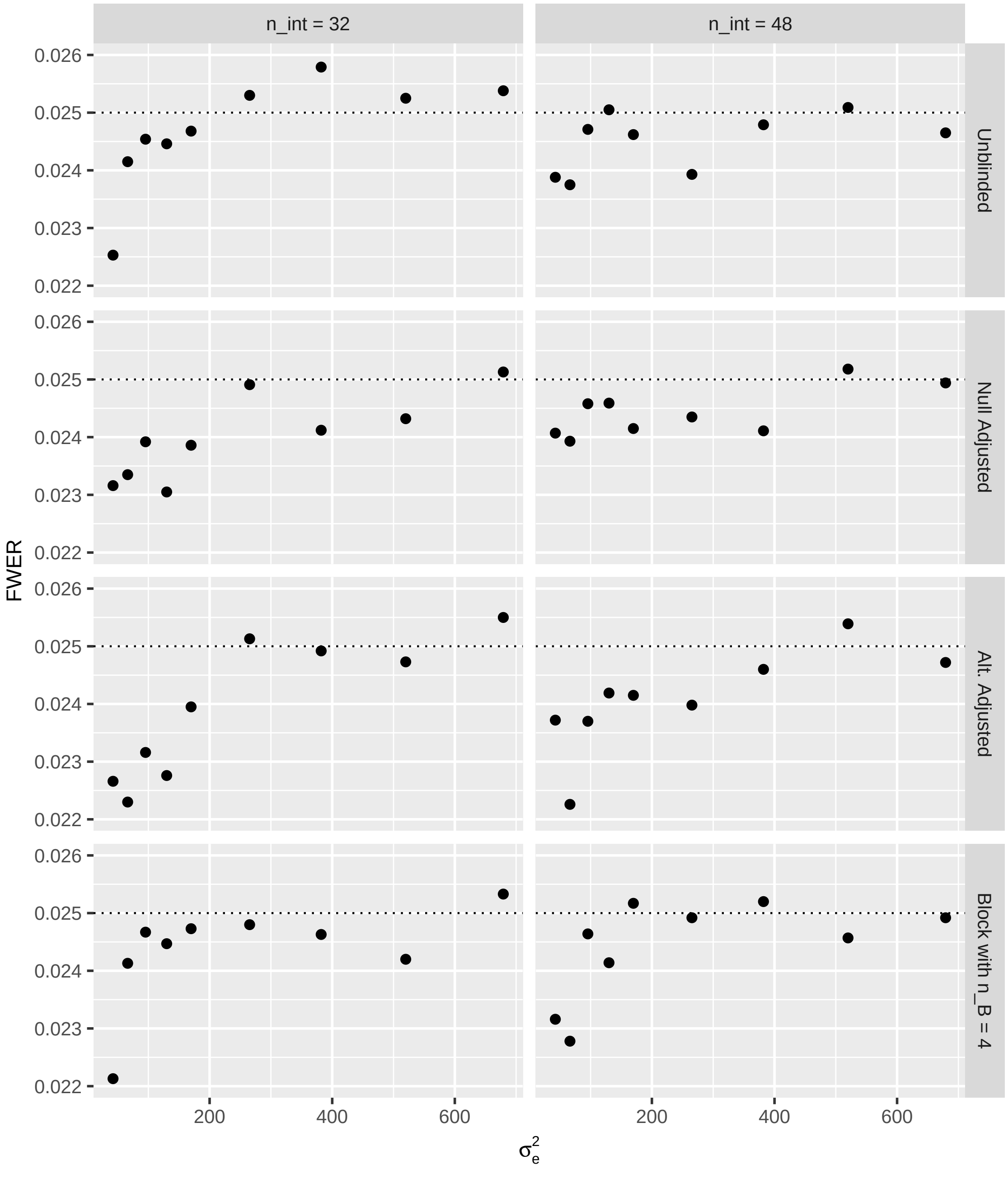

Supplementary Figure 21: The simulated familywise error-rate (FWER) is shown under the global null hypothesis for each of the re-estimation procedures when $n_{\text{int}} \in \{32, 48\}$, as a function of the within person variance σ_e^2, for Example 3. The Monte Carlo error is approximately 0.0005 in each instance. The dashed line indicates the desired value of the FWER.

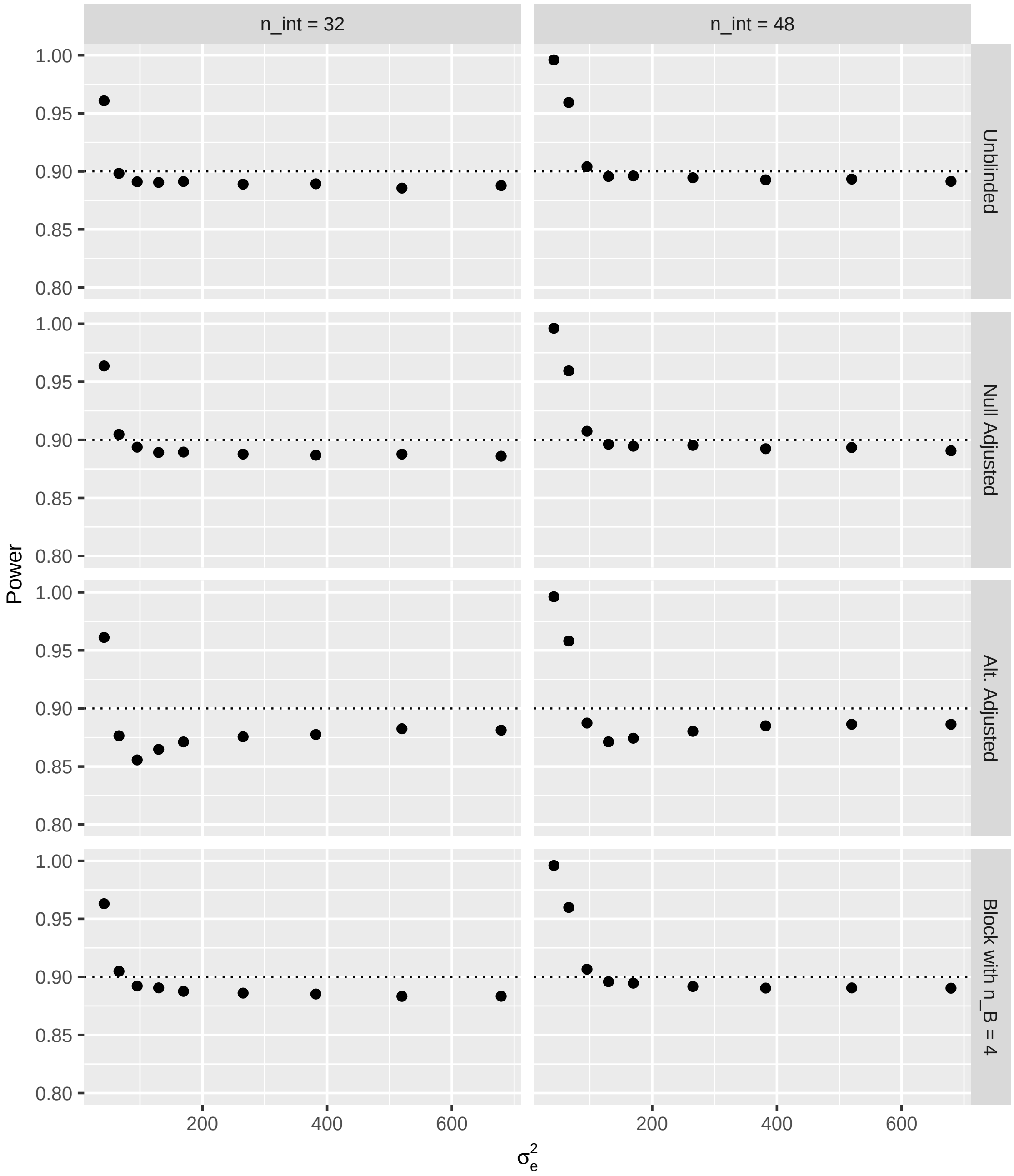

Supplementary Figure 22: The simulated power is shown under the global alternative hypothesis for each of the re-estimation procedures when $n_{\text{int}} \in \{32, 48\}$, as a function of the within person variance σ_e^2, for Example 3. The Monte Carlo error is approximately 0.001 in each instance. The dashed line indicates the desired value of the power.

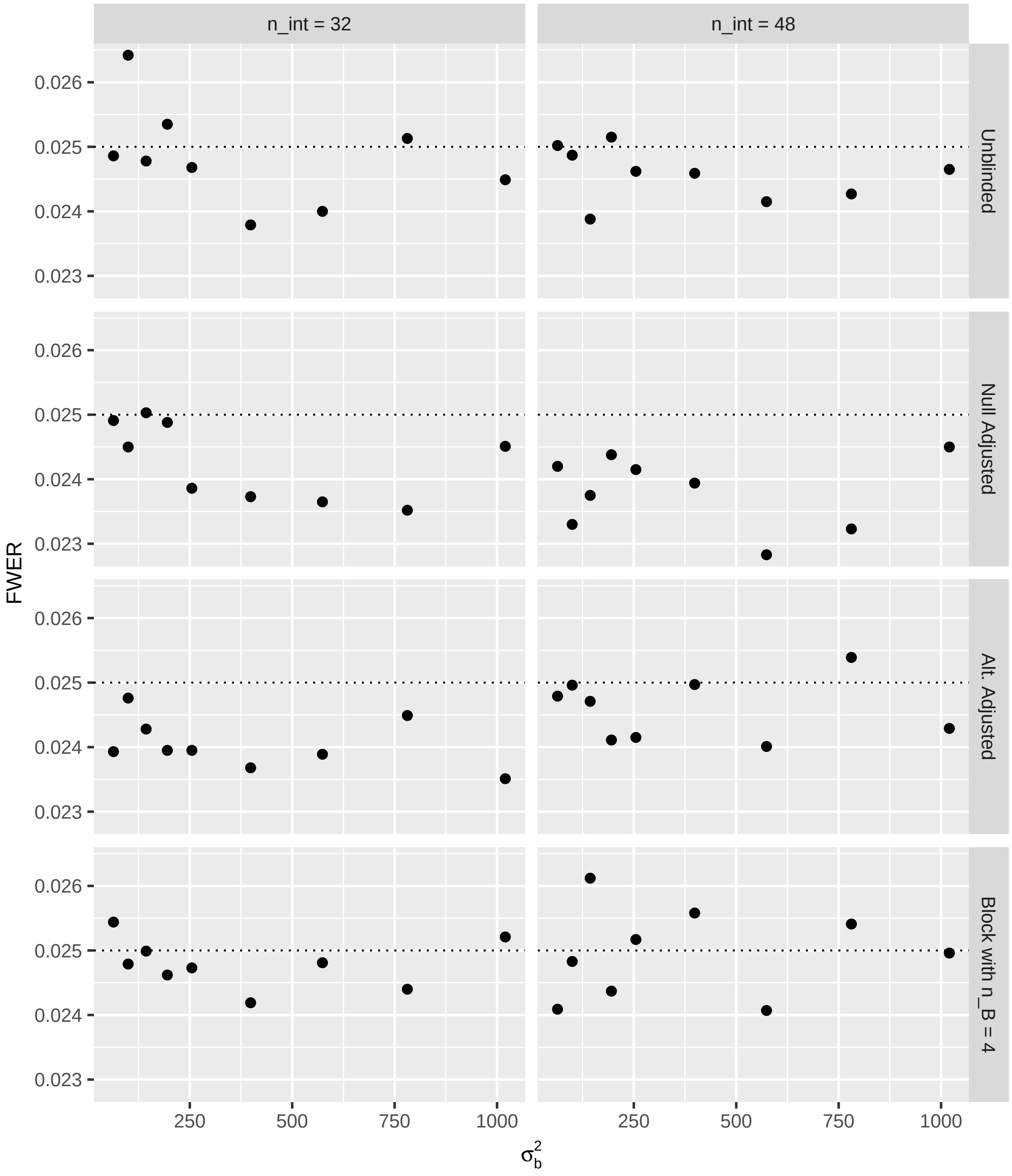

Supplementary Figure 23: The simulated familywise error-rate (FWER) is shown under the global null hypothesis for each of the re-estimation procedures when $n_{\text{int}} \in \{32, 48\}$, as a function of the within person variance σ_b^2, for Example 3. The Monte Carlo error is approximately 0.0005 in each instance. The dashed line indicates the desired value of the FWER.

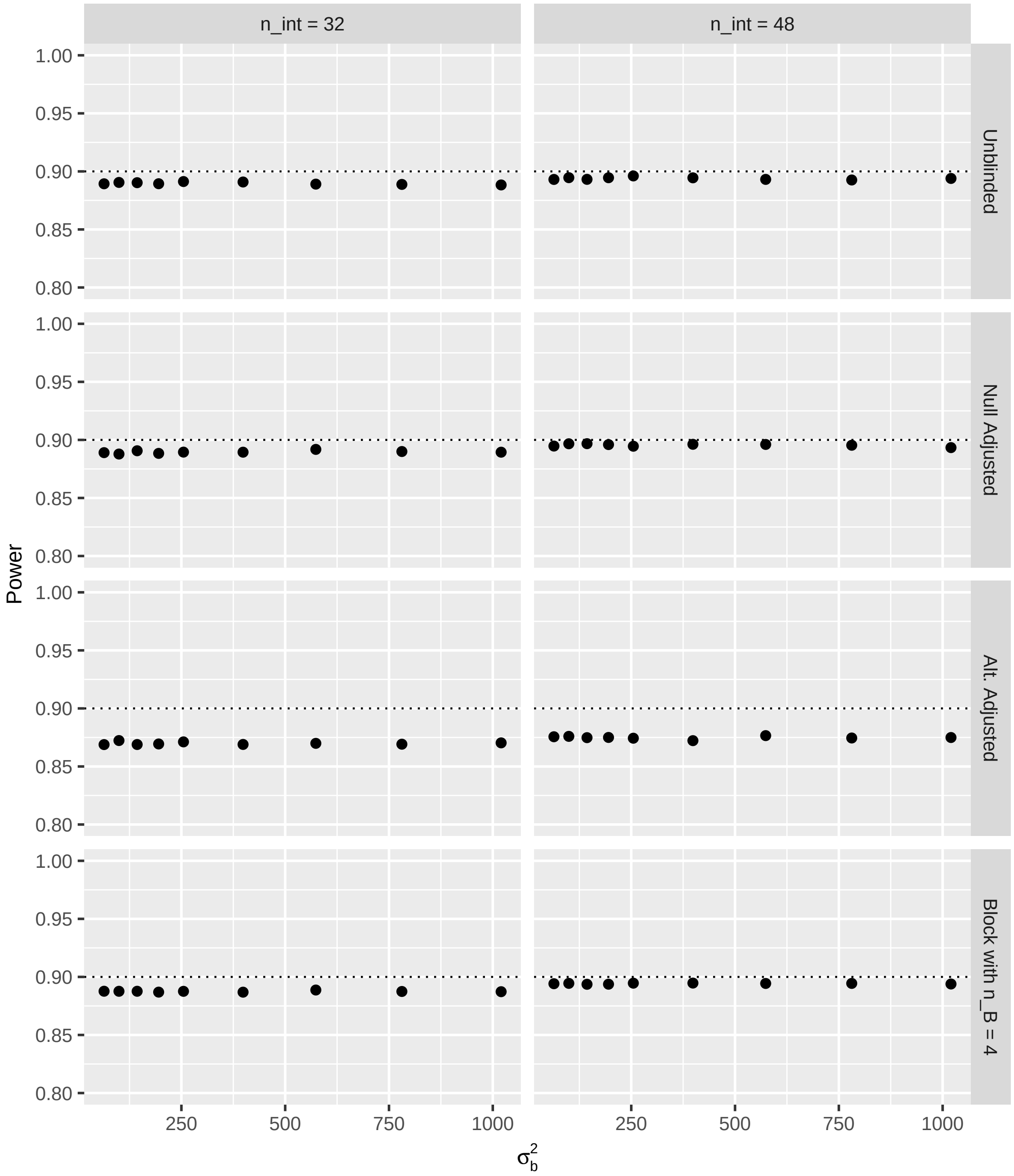

Supplementary Figure 24: The simulated power is shown under the global alternative hypothesis for each of the re-estimation procedures when $n_{\text{int}} \in \{32, 48\}$, as a function of the within person variance σ_b^2, for Example 3. The Monte Carlo error is approximately 0.001 in each instance. The dashed line indicates the desired value of the power.

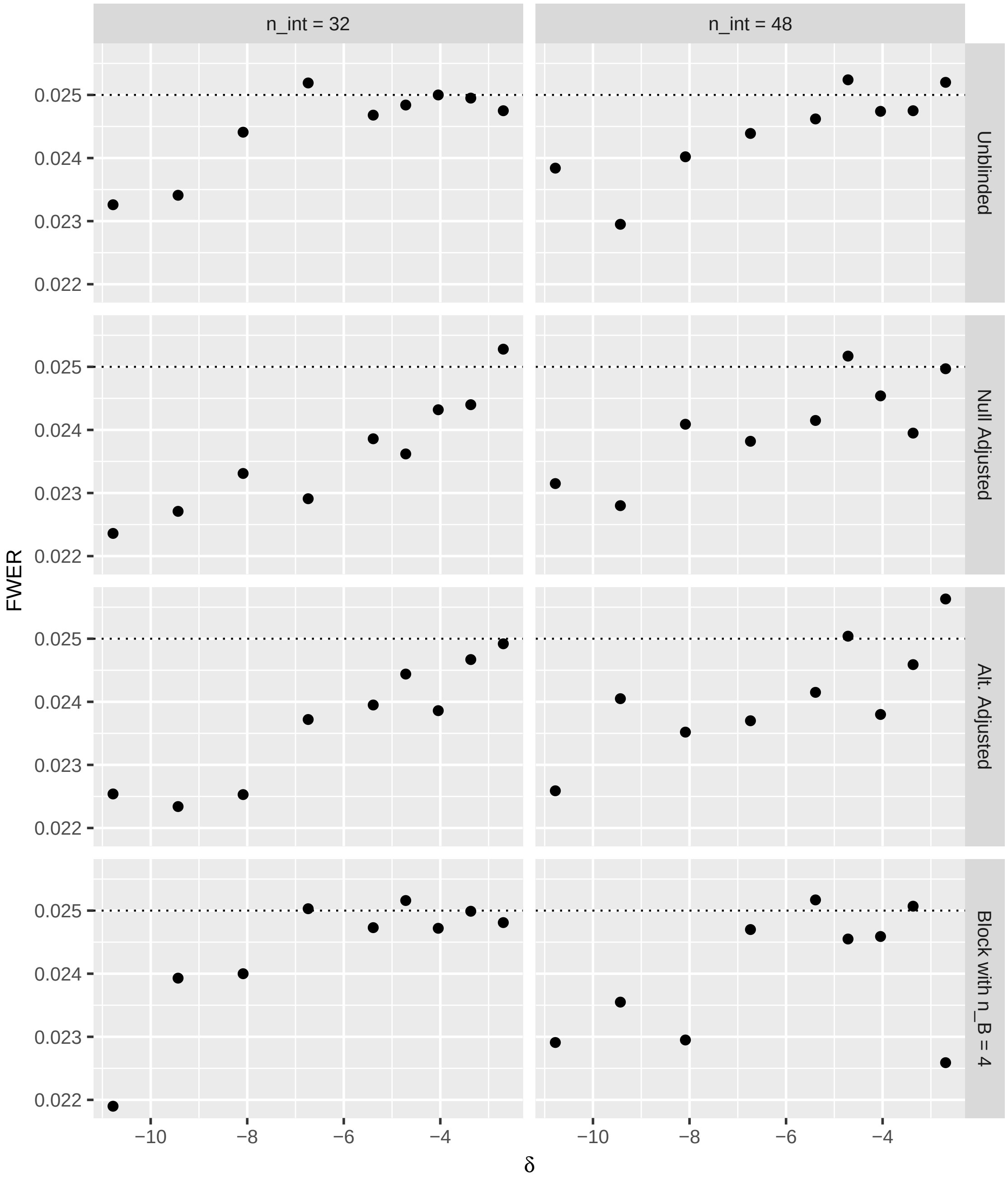

Supplementary Figure 25: The simulated familywise error-rate (FWER) is shown under the global null hypothesis for each of the re-estimation procedures when $n_{\text{int}} \in \{32, 48\}$, as a function of the clinically relevant difference δ, for Example 3. The Monte Carlo error is approximately 0.0005 in each instance. The dashed line indicates the desired value of the FWER.

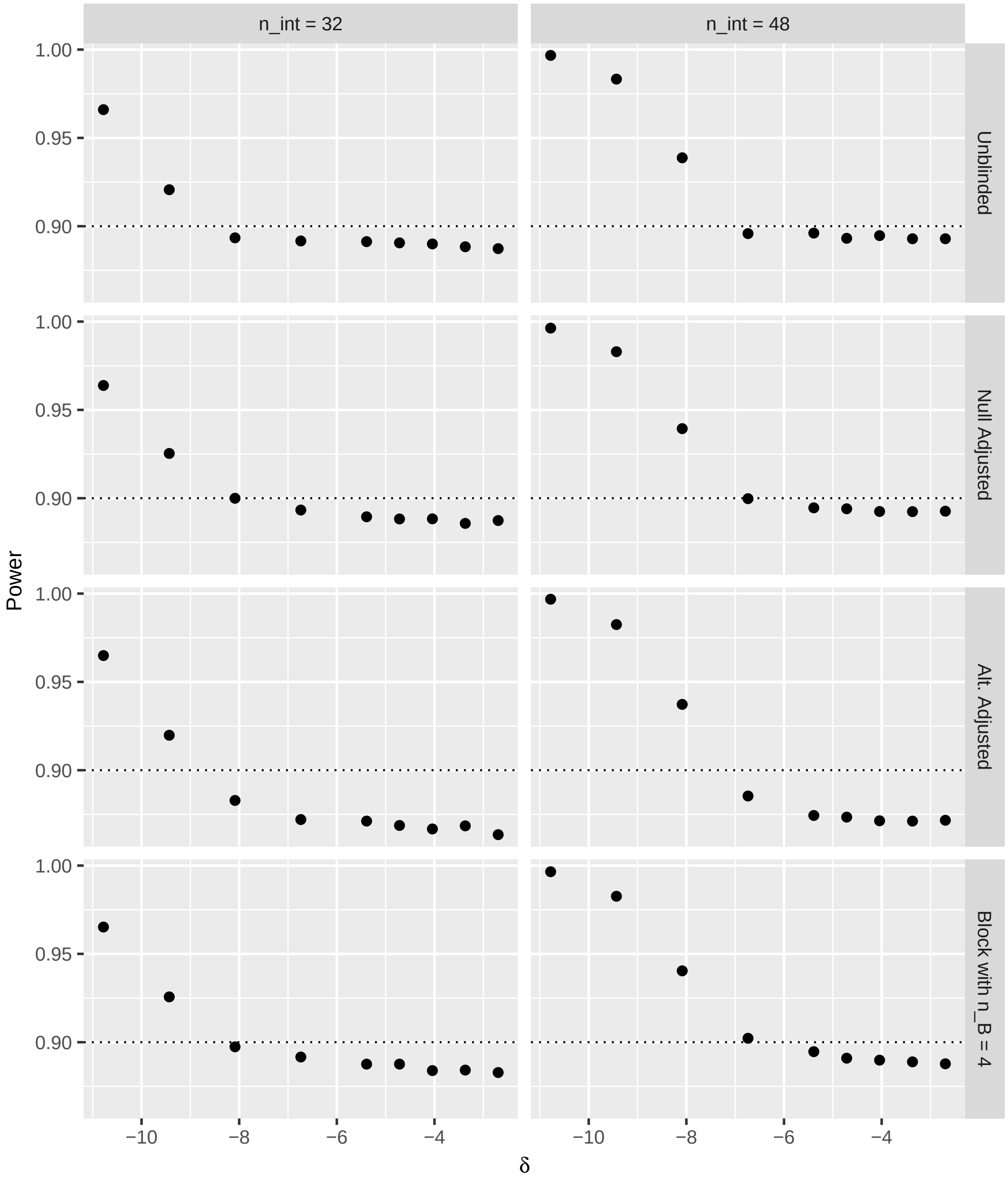

Supplementary Figure 26: The simulated power is shown under the global alternative hypothesis for each of the re-estimation procedures when $n_{\text{int}} \in \{32, 48\}$, as a function of the clinically relevant difference δ, for Example 3. The Monte Carlo error is approximately 0.001 in each instance. The dashed line indicates the desired value of the power.

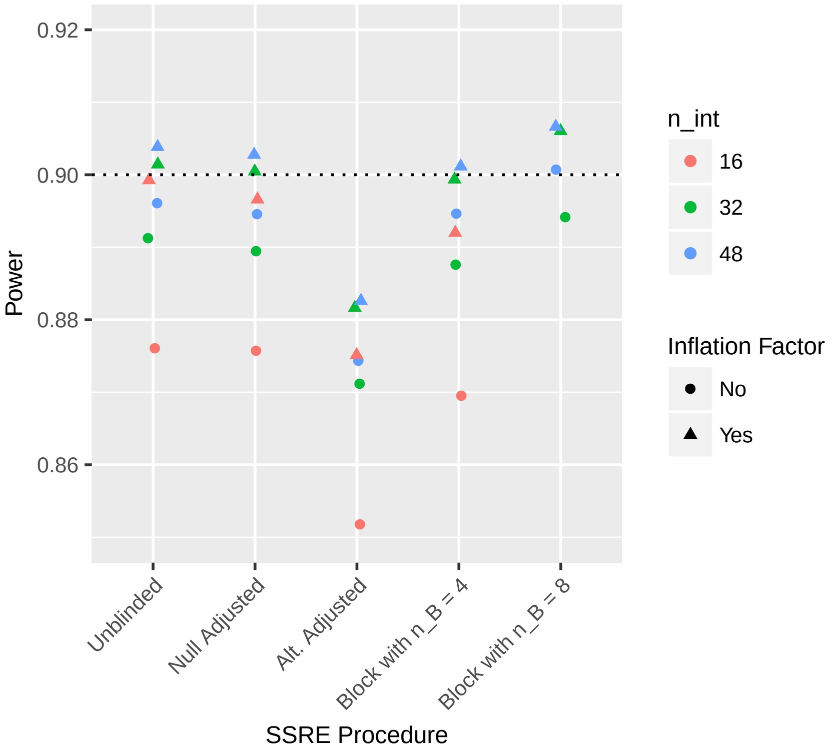

Supplementary Figure 27: The influence of the considered inflation factor upon the power of the re-estimation procedures under the global alternative hypothesis is shown for several values of n_{int}, for Example 3. The dashed line indicates the desired value of the power.
